# Stochastic expression of invasion genes in *Plasmodium falciparum* schizonts

**DOI:** 10.1038/s41467-022-30605-z

**Published:** 2022-05-30

**Authors:** Jaishree Tripathi, Lei Zhu, Sourav Nayak, Michal Stoklasa, Zbynek Bozdech

**Affiliations:** School of Biological Sciences, Nanyang Technological University, Singapore, 637551 Singapore

**Keywords:** Parasite genomics, Transcriptomics

## Abstract

Genetically identical cells are known to exhibit differential phenotypes in the same environmental conditions. These phenotypic variants are linked to transcriptional stochasticity and have been shown to contribute towards adaptive flexibility of a wide range of unicellular organisms. Here, we investigate transcriptional heterogeneity and stochastic gene expression in *Plasmodium falciparum* by performing the quasilinear multiple annealing and looping based amplification cycles (MALBAC) based amplification and single cell RNA sequencing of blood stage schizonts. Our data reveals significant transcriptional variations in the schizont stage with a distinct group of highly variable invasion gene transcripts being identified. Moreover, the data reflects several diversification processes including putative developmental “checkpoint”; transcriptomically distinct parasite sub-populations and transcriptional switches in variable gene families (*var*, *rifin*, *phist*). Most of these features of transcriptional variability are preserved in isogenic parasite cell populations (albeit with a lesser amplitude) suggesting a role of epigenetic factors in cell-to-cell transcriptional variations in human malaria parasites. Lastly, we apply quantitative RT-PCR and RNA-FISH approach and confirm stochastic expression of key invasion genes, such as, *msp1*, *msp3*, *msp7, eba181* and *ama1* which represent prime candidates for invasion-blocking vaccines.

## Abstract (web summary)

Genetically identical cells can be phenotypically diverse to allow adaptive flexibility in a given environment. This phenotypic diversity is driven by epigenetic and transcriptional variability. Here, Tripathi et al. perform scRNA-seq of isogenic and non-isogenic *Plasmodium falciparum* schizont populations to explore transcriptional heterogeneity and stochastic gene expression during the course of development.

## Introduction

Malaria, an infectious disease caused by *Plasmodium* parasites, remains a threat to human health with 228 million cases and 405,000 deaths recorded in 2018^1^. Humans can be infected by six different *Plasmodium* species, *P. vivax, P. falciparum, P. ovale, P. malariae, P. knowlesi* and *P. cynomolgi*, with falciparum malaria being the most virulent. During its complex life cycle, *Plasmodium* parasites differentiate into multiple developmental stages, switching between sexual and asexual forms in their primary (mosquito) and intermediate (human) host respectively. Throughout this journey, the parasite thrives in different host environments, selection pressures (such as, host immune response, exposure to antimalarials) and sudden perturbations. This requires remarkable phenotypic plasticity for a single cellular eukaryotic pathogen to survive. Previously, phenotypic plasticity has been demonstrated in *Plasmodium* parasites in relation to sexual conversion rates and ratio, asexual invasion rates and burst size^2^. Within-host environmental factors such as, anaemia, drug treatment, nutritional status, circadian rhythm and age of red blood cells (RBCs) available for invasion have been shown to modulate parasite replicative and reproductive efforts^3–8^. However, the molecular basis underpinning this phenotypic variability still remains uncharacterised.

Phenotypic diversity has been shown to be driven by epigenetic and transcriptional variability in other systems^9,10^. For example, studies on bacteria and yeast^11–13^ has demonstrated that cells with multiple ‘physiological states’ can exist in a genetically identical population^14^. In the context of *Plasmodium* specifically, clonal variation in the expression of members of surface antigen gene families such as *var*, *rifin* and *stevor* is well-known^15–17^. Similarly, expression of solute transport channels, *clag3.1* (PF3D7_0302500) and *clag3.2* (PF3D7_0302200), on the surface of parasitized RBCs is found to be mutually exclusive in isogenic clones^18,19^. *pfap2-g* (PF3D7_1222600), the master regulator of sexual differentiation, is another gene known to be associated with heterochromatin protein 1 (HP1) and exhibits clonally variant expression^20,21^. These studies confirm the presence of non-genetic transcriptional heterogeneity in *Plasmodium* parasites and calls for a systematic transcriptome-wide investigation of gene expression variability.

One of the latest cutting edge methodologies to study cell-to-cell transcriptional variability and detect highly variable genes (HVGs) in a homogenous cellular population is single cell RNA sequencing (scRNAseq)^22–25^. In the context of malaria, published scRNAseq studies collectively describe transcriptional profiles associated with sexual commitment, development of various *P. falciparum* and *P. berghei* stages (i.e., liver schizonts, ookinetes, sporozoites, blood stages) as well as gene expression patterns of *P. vivax* isolates from non-human primate blood stream infections^26–32^. Undoubtedly, this has provided valuable insights into *Plasmodium* biology, however, up until now, experimentally validated stochastic gene expression studies within individual parasite forms (developmental stages) remain to be reported. A key requirement for confident detection of transcriptional stochasticity is a reliable whole transcriptome amplification (WTA) method. In this work, we optimise the quasilinear multiple annealing and looping based amplification cycles (MALBAC) to suit the lower RNA content in *P. falciparum* asexual stages (compared to mammalian cells) and minimize cell-to-cell technical variation as compared to PCR-based WTA used in previous studies. Using this technique, we generate single cell transcriptomes of two cohorts of *P. falciparum* schizonts originating from non-isogenic and isogenic parasite populations, respectively. Here, we demonstrate several “layers” of transcriptional variations linked to differential life cycle progression, single cell transcriptional subpopulations (SCTS) and stochastic transcriptional variability of genes (HVGs) involved in crucial cellular processes such as invasion (and others). We also characterize differential transcriptional patterns for key hypervariable gene families such as *var*, *rifin* and *phist* involved in immune evasion and host-parasite interactions^15,33–36^. Last but not the least, we provide experimental validation of cell-to-cell transcriptional variability of several previously unidentified HVGs, which supports the validity of the derived technique for confident characterizations of stochastic gene expression in malaria parasites.

## Results

### Multiple annealing and looping based amplification cycles (MALBAC) for single cell transcriptomes from *P. falciparum* Schizonts

**Fig. 1:**
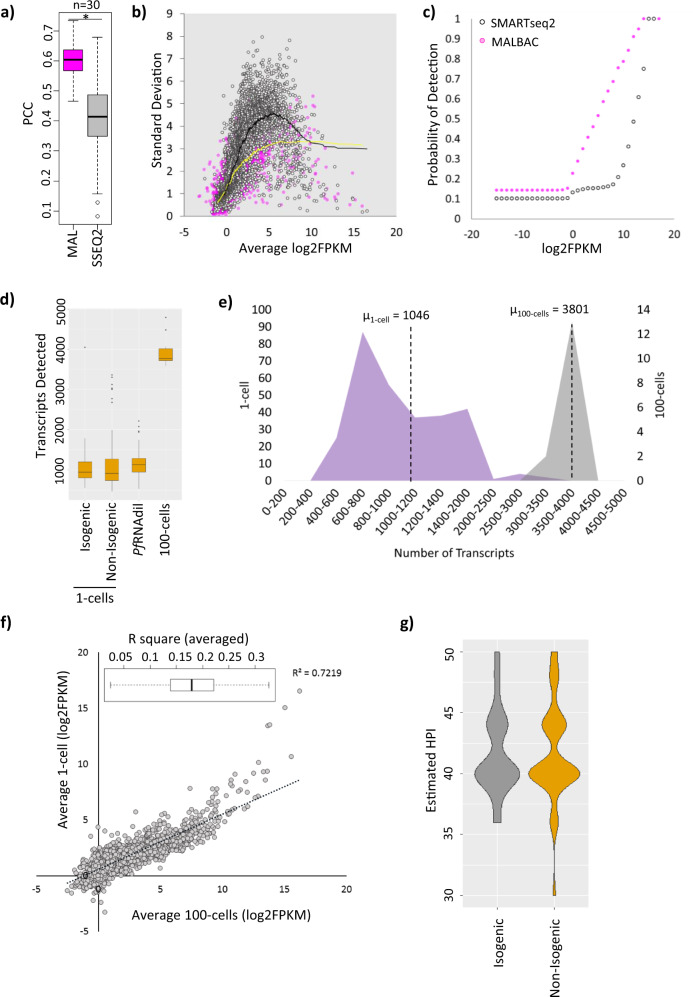
Highly reproducible MALBAC-based amplification of single cell transcriptomes from *P. falciparum* schizonts. **a** A boxplot showing Pearson correlation coefficients for PfRNAdil (*n* = 30) amplified by SMARTseq2 (SSEQ2) or MALBAC (MAL). Asterisk denotes significant Wilcoxon rank sum test *p*-value (*p* < 2.2e-16). **b** Scatter plot depicting relationship between standard deviation (SD) and mean transcript expression (log2FPKM) across PfRNAdil replicates (*n* = 30) for the two techniques. **c** A scatter plot showing the probability of detection of transcripts (detected in atleast 50% PfRNAdil) across PfRNAdil replicates (*n* = 30) amplified by MALBAC or SMARTseq2. **d** A boxplot showing number of transcripts detected in 1-cell (isogenic (*n* = 97) and non-isogenic (*n* = 295)), 100-cells (*n* = 15) and PfRNAdil (*n* = 49) samples. **e** A frequency distribution plot of number of transcripts detected in 1-schizonts and 100-schizonts samples. *µ* denotes the mean number of transcripts expressed in the 1-cell and 100-cells group. **f** Correlation between averaged 1-cell and 100-cell transcriptomes. The range of averaged R^2^ values for individual 1-cell and 100-cell transcriptome correlations is shown as a boxplot (inset, median and standard deviation shown, *n* = 295 for 1-cell, *n* = 15 for 100-cells). **g** A violin plot depicting estimated age distribution of non-isogenic and isogenic schizonts. Source data are provided as a Source Data file. All box plots show centre line as median, box limits as upper and lower quartiles, whiskers as minimum to maximum values.

Here, we wished to develop a WTA strategy for scRNAseq analysis of the human malaria parasite *P. falciparum* that is based on quasilinear amplification to achieve broad, uniform and quantitatively reproducible transcriptome coverage. The goal was to significantly improve detections of genuine cell-to-cell (stochastic) gene expression variations within otherwise morphologically uniform parasite populations, such as a single developmental stage. The rationale is to minimize extensive PCR cycling that could reduce the complexity of the final cDNA library by amplification bias^37–39^ and is known to be the main limitation of the standard techniques applied in previous studies^26–30,32^. With this rationale, we optimised a quasilinear amplification method MALBAC^39,40^ for WTA of *P. falciparum* schizonts, and compared this to an in-house optimised version of PCR-based SMARTseq2 method^41,42^. For this, thirty RNA dilutions prepared from triplicates of 10 schizonts diluted 10-folds each to achieve an equivalent amount of total RNA of a single cell (PfRNAdil) were amplified using both MALBAC and SMARTseq2. As shown in Fig. 1a, PfRNAdil transcriptomes generated by MALBAC showed significantly higher correlation (pairwise Pearson Correlation Coefficient, PCC, Wilcoxon rank sum *p* value < 2.2e–16) relative to SMARTseq2. Similarly, MALBAC amplified PfRNAdil exhibited lower standard deviation (SD) in gene expression across replicates with lower SD dependency on the overall mean transcript abundance. This suggests that quasilinear amplification introduced lesser technical variation compared to exponential amplification (Fig. 1b). MALBAC also performed superior to SMARTseq2 in transcript detection as we observed a higher probability of low abundance transcript detection with MALBAC (Fig. 1c). Overall, these findings suggest that MALBAC is a more sensitive and reproducible method and is more suitable technique for the intended study. 1-cell and 10-cell transcriptomes generated by the two WTA methods also showed similar trends with higher correlations between 1-cell (*R*^2^ = 0.369) and 10-cells (*R*^2^ = 0.646) transcriptome triplicates, lower SD and higher probability of transcript detection observed for MALBAC (Supplementary Fig. 1). This demonstrates that MALBAC captures 1-cell and 10-cell transcriptomes with higher sensitivity and quantitative reproducibility across a wide range of transcript abundance, making it most suitable to analyse cell-to-cell gene expression variability.

Next, we applied MALBAC to generate single cell transcriptomes of *P. falciparum* schizonts collected from non-isogenic and isogenic parasite populations. Briefly, using fluorescence-assisted cell sorting (FACS) we isolated 300 individual schizonts from a highly synchronized culture of the *P. falciparum* 3D7MR4 strain; representing a non-isogenic population. Subsequently, we generated an isogenic clonal culture by serial dilution of the 3D7MR4 parasites and collected 100 individual schizonts after fourteen generations of clonal expansion (presumably from a single founding cell). The rationale behind the comparison of non-isogenic and isogenic schizonts was to dissect out contribution of genetic differences and/or epigenetic factors towards transcriptional variability in a population. Overall, we detected 5488 transcripts with 2695 being detected in more than 10% of the non-isogenic cells (Supplementary Fig. 2). Similar transcriptome coverage was achieved with the isogenic group of parasites with 5260 transcripts detectable in total. The mean number of transcripts detected per schizont was ~1040 in both isogenic and non-isogenic cohorts as compared to ~3800 transcripts detected in 100-cells bulk control (*n* = 15) (Fig. 1d, e). This is consistent with the transcriptome generated from samples in which RNA obtained from 10 schizonts was diluted 10-fold to achieve an equivalent amount of total RNA of a single cell (PfRNAdil). Here, we detected a mean of 1145 transcripts per PfRNAdil (*n* = 49) (Fig. 1d). This represents a significant improvement to the previous studies in which mean/median of genes detected per schizont ranged between 212 to 938^26,27,29^. To assess the quality of the MALBAC-generated results, we correlated the average of all 1-cell and 100-cells transcriptomes achieving an *R*^2^ value of 0.72, thus confirming that the gene expression signal amplified by MALBAC represents true biological signal as detected in bulk samples (Fig. 1f). The *R*^2^ values correlating individual 1-cell and 100-cells transcriptomes ranged from 0.02 to 0.33 (Fig. 1f, top left inset). Lastly, majority of the sequenced 1-cell transcriptomes were estimated to be between 40–44 hour post invasion (HPI) (see below) by correlation to a high-resolution bulk 3D7 reference transcriptome ^42^ (Fig. 1g), thus confirming successful isolation and amplification of late asexual stages.

### Schizont subpopulations with unique transcriptional profiles identified in *P. falciparum* parasites

**Fig. 2:**
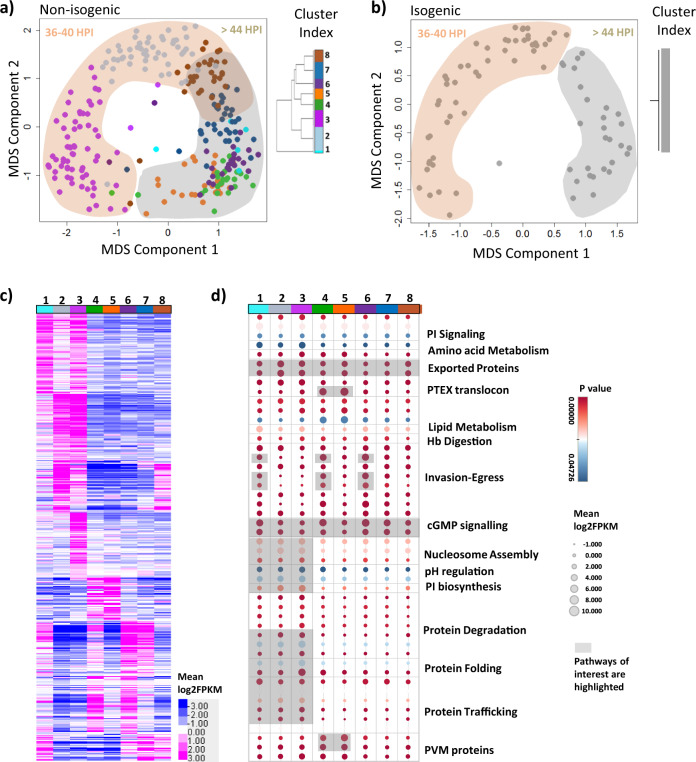
Schizont subpopulations with unique transcriptional profiles identified in non-isogenic *P. falciparum* parasites. A multidimensional scaling (MDS) plot showing first two components (MDS1 and MDS2) depicting maximum differences between individual schizonts in **a** non-isogenic and **b** isogenic group. RSEC-based SCTS hierarchy is shown in right hand inset (for both (**a**, **b**)) with each SCTS represented by a different colour for non-isogenic schizonts. The two major age groups (36-40 HPI and >44 HPI) identified has been overlaid on the MDS plot. **c** A heatmap showing average expression of differentially expressed (DE) genes (mean normalised log2FPKM) in each SCTS of non-isogenic parasites. **d** A dot plot of various functional pathways enriched in non-isogenic parasite SCTS. The colour and size of each circle depicts *p*-value and mean log2FPKM value respectively with pathways of interest highlighted in grey. Source data are provided as a Source Data file.

First, we interrogated the generated dataset for the presence of parasite sub-populations that reflect transcriptional variability at a broader (supracellular) level. Applying a custom designed data quality filter (refer to *Methods*), we select 271 non-isogenic and 79 isogenic 1-cell transcriptomes and subjected these to data normalization, dimensionality reduction and removal of batch effects (Supplementary Fig. 3) by ZINB-WaVE modelling as described^43^. As shown in Figs. 2a, b, the first two components of the multidimensional scaling (MDS) account for the maximum differences between 1-cell transcriptomes of both non-isogenic and isogenic parasites. This MDS-based stratification aligned well with the parasite age differentiation (described in Fig. 1f) with a fraction of cells falling into the 36–40 and >44 HPI groups, respectively (Fig. 2a, b). As expected, similar HPI categorization could be deciphered for the non-isogenic as well as the isogenic population with comparable distribution patterns. Subsequently, we applied resampling-based sequential ensemble clustering (RSEC) to evaluate a possible finer substructure within each dataset that would indicate the existence of distinct subpopulations. Interestingly, eight subpopulations were detected in the non-isogenic parasite group each defined by a specific transcriptional profile (termed as Single-Cell Transcriptional Subpopulations, SCTS) (Fig. 2a). The pseudotemporal ordering followed the estimated age progression of schizonts with SCTS 2, 3 and 8 falling onto ~36 to 40 HPI and SCTS 1,4,5,6 and 7 onto ≥ 44 HPI groups (Fig. 2a and Supplementary Fig. 4). Interestingly, for the isogenic 1-cell population, identical RSEC clustering parameters did not detect any subpopulations (Fig. 2b). To account for sampling size difference between non-isogenic and isogenic schizonts, we repeated the RSEC clustering on both isogenic and non-isogenic parasites using 428 highly representative genes (refer to *Methods*) after sub-sampling of 80 single non-isogenic schizonts. For isogenic schizonts, cluster merging was observed at a differential gene expression (DE) cut off of >25% between clusters (Supplementary Fig. 5a). However, when 80 non-isogenic schizonts were sub-sampled randomly, multiple SCTS could still be identified majority of the times (62% confidence, Supplementary Fig. 5b). This suggests that while the HPI (age) separation reflects intrinsic properties of schizont maturation of any given schizont population, the SCTSs represent a deeper differentiation of genetically (or otherwise) more diverse parasite cohorts.

Next, we applied the local regression method loess and identified 440 genes exhibiting differential expression along the pseudotemporal progression defining the eight identified SCTS (Fig. 2c and Supplementary Data 1). Pathway enrichment analyses using the Malaria Parasite Metabolic Pathways (MPM) database showed that each SCTS carries a unique set of biological functions/pathways represented by the differentially expressed genes (Supplementary Data 2)^44^. As shown in Fig. 2d, protein folding, COPI/COPII-mediated vesicular trafficking and protein degradation pathways generally showed higher average expression in the SCTS 1, 2 and 3. This included genes encoding for, coatomer subunits (α,β,γ), endoplasmic reticulum chaperone GRP170 and E3-ubiquitin ligase, etc. Interestingly, ubiquitin activation was recently shown to be essential for schizonts maturation^45^. Similarly, expression of pH regulation, nucleosome assembly and phosphatidyl inositol biosynthesis-related pathways was also higher in SCTS 1, 2 and 3. This may be indicative of organisation of newly replicated DNA and active membrane biogenesis concomitant with the segregation of daughter parasites (merozoites) during mid-late stage schizogony^46^. On the other hand, expression of parasitophorous vacuole membrane associated proteins pathway and PTEX translocon pathway involved in protein export, was found to be highest in SCTS 4 and 5 mainly consisting of late stage schizonts (i.e. >44 HPI). In particular, pathways linked to invasion and exported proteins showed variable expression levels between SCTSs 1,4,5,6 and 7 belonging to similar pseudotime interval (Fig. 2d and Supplementary Fig. 6). For instance, SCTS 1, 4 and 6 showed higher average expression of pathways associated with invasion ligands for erythrocyte receptors, merozoite surface proteins and motility, represented by genes encoding for, erythrocyte binding antigen 175 (PF3D7_0731500 or *eba175*), merozoite surface protein 5 (PF3D7_0206900 or *msp5*) and merozoite surface protein 6 (PF3D7_1035500 or *msp6*), and reticulocyte binding protein homologues. SCTS 5 (also 44 HPI), on the other hand, showed lower average expression of the above-mentioned invasion pathways. cGMP signalling pathway involved in invasion and egress was uniformly highly expressed across all SCTSs with only subtle variations. Taken together, these results suggest the existence of multiple transcriptional ‘physiological states’ established within a single parasite population, independent of developmental state/age groups, over extended periods of cell diversifications (here prolonged in vitro culturing over multiple generations). The SCTS may represent subtle but significant diversion of the schizont maturation process with a putative checkpoint at approximately 44 HPI, all of which may contribute to phenotypic plasticity of malaria parasite prior and during the invasion process.

To assess the relationship between the SCTS-driving transcriptional variation and genetic diversity, we identified 284 single nucleotide polymorphisms (SNPs) represented in 50% of the isogenic or non-isogenic schizonts population and were used for the analysis of the genetic population structure (refer to *Methods*). The majority of these 284 SNPs occurred at a low allele frequency in both schizonts groups (Supplementary Fig. 7a). Upon further relaxing of the stringency criteria, 3459 SNPs could be detected however majority of these exhibited very low representation rate in the population and were excluded from the analysis (Supplementary Fig. 8a). A Principal Component Analysis (PCA) using the highly represented 284 SNPs revealed that the 271 non-isogenic cells segregated into three clusters by the top two principal components (PCs), whereas the 79 isogenic cells overlaid with only two of these clusters suggesting that the founding cell of the isogenic population originated from either of those clusters (Supplementary Fig. 7c). Interestingly, the distribution of SCTS 1,3,4 and 6 showed bias to one of the three genetic clusters for non-isogenic parasites (hypergeometric *p* < 0.0001), suggesting that genetic factors may partially drive and/or underlie transcriptional differentiation of these sub-populations (Supplementary Fig. 7d). All other SCTSs showed similar distribution across the three genetic clusters indicating that the observed transcriptional differentiation is not driven by the (exonic) SNP profile of those parasites. However, we cannot exclude the possibility that other more subtle genetic variations in other regions (such as promoter/intergenic regions) that were beyond detection of the scRNAseq analysis may interline these transcriptional subpopulations. Notably, several of the SNPs showed concentration in a small number of genes involved in host-parasite interactions (Supplementary Fig. 8b and Supplementary Data 8).

### Stochastic expression of highly variable genes in *P. falciparum* schizonts

**Fig. 3:**
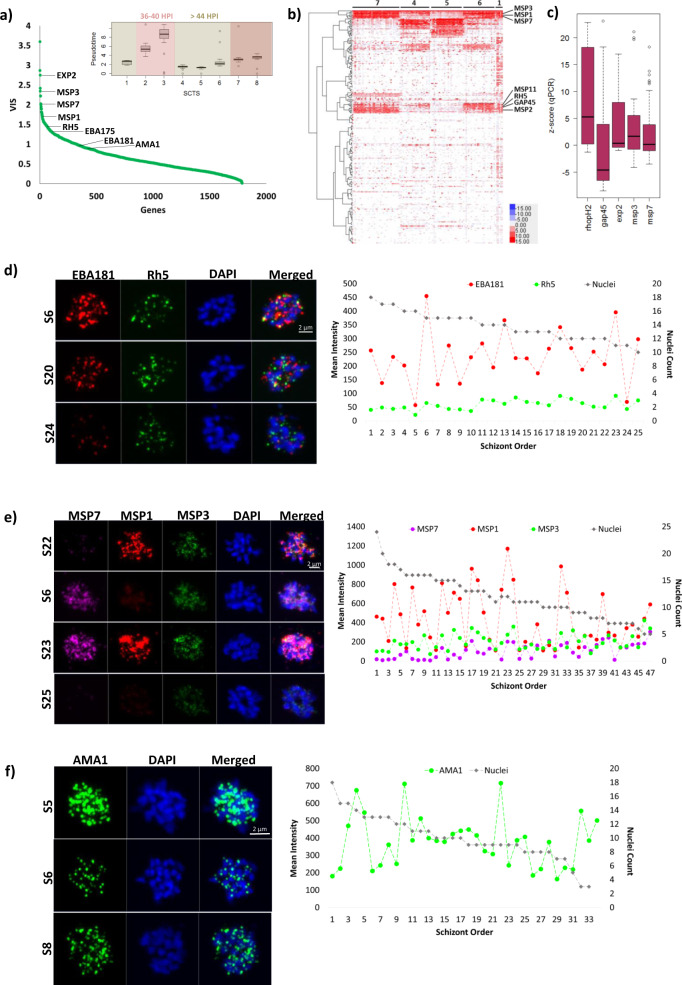
Stochastic Gene Expression in *P. falciparum* Schizonts. **a** A graph showing variability index score (VIS) of individual genes in non-isogenic schizonts (*n* = 271 1-cell) belonging to similar pseudotime interval (i.e. SCTS 1,4,5,6,7 (*n* = 118 1-cell) in inset) with key invasion genes labelled. Only genes expressed in more than 10 PfRNAdil were considered to avoid artificially inflated VIS. **b** A heatmap showing expression of HVGs (log2FPKM) in non-isogenic schizonts with genes of interest labelled. Higher and lower expression (log2FPKM) is shown in red and blue respectively. **c** A boxplot showing z-score calculated from Ct values for each gene transcript measured in single schizonts (*n* = 16 for *rhop2*, *exp2;* *n* = 38 for *msp3, msp7, gap45*) and *Pf*RNAdil by qRT-PCR. Median Ct is shown as black line. Primer sequences are provided in Supplementary Data 6. Confocal microscopy images and line graphs showing transcript levels of **d** *eba181*, *rh5*, **e** *msp1, msp3, msp7* and **f** *ama1*, in individual schizonts. Representative images of single 3D7MR4 schizonts stained for **d** *eba181* (red) and *rh5* (green), and, **e** *msp1* (red), *msp3* (green) and *msp7* (purple) and **f** *ama1* (green) transcripts using customised RNA probes are shown on the left. S6, S20, S24 and S6, S22, S23, S25 and S5, S6, S8 refers to the schizont order in the line graph for **d**–**f** respectively for which the image is shown. Parasite nuclei were stained with DAPI. Between 30 to 50 schizonts were imaged from one RNA-FISH experiment for each combination of probes. Line graphs on the right-hand side show the mean intensity quantified for each gene transcript in individual parasites using the ZEN 3.0 (blue edition ZEN lite) software. Source data are provided as a Source Data file. All box plots show centre line as median, box limits as upper and lower quartiles, whiskers as minimum to maximum values.

Further inspection of our data suggested a presence of a small but distinct set of genes with fundamentally broader transcriptional differences indicating stochastic mRNA variations. To study this further, we quantified transcriptional variability of individual genes by assigning a variability index score (VIS) calculated as the SD of z-score per gene with respect to PfRNAdil (Fig. 3a). To circumvent transcriptional variability arising from age/developmental differences between individual cells, we focused on analyzing the late stage schizonts (i.e. ~44 HPI group consisting of SCTS 1, 4, 5, 6, 7) which belonged to a common pseudotime interval (Fig. 3a inset and Supplementary Fig. 6). In this group of schizonts, several invasion genes such as *msp1* (PF3D7_0930300)*, msp3* (PF3D7_1035400)*, msp7* (PF3D7_1335100)*, rh5* (PF3D7_0424100)*, eba175, eba181* (PF3D7_0102500) and *ama1* (PF3D7_1133400) exhibited a high VIS (Fig. 3a). This suggests variable level of expression of crucial invasion gene transcripts in mature schizonts at similar developmental stage. In addition to the invasion genes, we found genes involved in other essential cellular functions such as nutrient/protein export (PF3D7_1471100 or exported protein 2), organelle segregation and cytokinesis (PF3D7_1246200 or actin I) and egress (PF3D7_1337800 or calcium dependent kinases 5), also exhibited high VIS (Supplementary Data 3).

The functional analyses of the SCTS (previous section) indicated that genes involved in the merozoite invasion exhibit particularly high levels of cell-to-cell variation at the schizont stage. To identify statistically significant HVGs, we applied the F-test analysis comparing 1-cells versus PfRNAdil (*p* < 0.05 and SD 1-cell>SD PfRNADil) for schizonts belonging to SCTS 1,4,5,6 and 7 (with similar pseudotime interval) to determine stochastic gene expression amongst 1-cells compared to PfRNAdils^47^ (Fig. 3b and Supplementary Fig. 6). As shown in Fig. 3b, invasion genes previously found to have high VIS (such as *msp1, msp3, msp7*, *rh5*) exhibited stochastic expression amongst late stage non-isogenic schizonts (Supplementary Data 4). A similar trend was observed in the isogenic schizonts as well, where invasion genes encoding for rhoptry associated proteins (*rap1* (PF3D7_1410400)*, rap2* (PF3D7_0501600), *rap3* (PF3D7_0501500)*, rhopH2* (PF3D7_0929400)*, rhopH3* (PF3D7_0905400)) and merozoites surface proteins (*msp1, msp3, msp4* (PF3D7_0207000), *msp* (PF3D7_1035600)) were found to be highly variable within the 40 HPI and/or 44 HPI age groups (Supplementary Data 5). Crucially, genes such as *msp1, msp3*, *msp7* and *gap45* (PF3D7_1222700) were found to be highly variable in both the non-isogenic and isogenic schizonts.

In addition to invasion related genes, genes involved in diverse cellular functions, such as, exported protein 2 (*exp2*), histone 4 (PF3D7_1105000), actin I, folate transporter 1 (PF3D7_0828600), ring exported protein 1 (PF3D7_0935900) and ring exported protein 3 (PF3D7_0936300) were also highly variable between single non-isogenic schizonts (Supplementary Data 4). Similarly, non-invasion genes presenting somewhat different set of functionalities such as erythrocyte vesicle protein 1 (PF3D7_0410000), malate dehydrogenase (PF3D7_0618500), peroxiredoxin (PF3D7_1027300), endoplasmic reticulum calcium binding protein (PF3D7_1108600 or *pferc*), calcium dependent protein kinase 1 (PF3D7_0217500 or *cdpk1*), etc. showed highly variable expression amongst isogenic schizonts (Supplementary Data 5). Interestingly, two of the HVGs in our dataset, i.e. AP2 transcription factor (PF3D7_0420300), and, a putative long chain polyunsaturated fatty acid elongation enzyme (PF3D7_0605900) were also previously reported to have variable expression levels in bulk RNA sequencing of 3D7 schizonts^48^. Taken together, here we identified a small but significant group of genes with fundamentally higher levels of cell-to-cell transcriptional variations that is far beyond differential mRNA levels underlying the HPI projections (Supplementary Fig. 9). Enrichment of invasion genes in this category (along with other functionalities) suggests that maintaining stochastic expression of these genes is crucial (hence conserved) presumably to ensure successful productive invasion across variable host cell surface receptor repertoire/environments. Maintenance of HVGs even within the isogenic population (presumably devoid of fundamental genetic variations) suggests a role of epigenetic factors in these processes. Indeed, the presence of multiple *var* genes, *rifins* and *surfin*s amongst the HVGs in non-isogenic schizonts only supports this concept given their association with a key epigenetic factor HP1, characterizing heterochromatin regions of the *P. falciparum* genome^49^.

### RNA-FISH and RT-PCR validations of highly variable gene expression in individual schizonts

To gain a stronger confidence in the derived MALBAC method, we experimentally validated the detected stochastic expression for a subset of the HVGs using gold standard method RNA fluorescence in situ hybridization (RNA-FISH) and quantitative reverse transcription PCR (qRT-PCR). The HVG status for *msp3, msp7, gap45*, *rhop2* and *exp2* was confirmed by qRT-PCR and/or RNA-FISH (Fig. 3c and Supplementary Fig. 10). Next, RNA-FISH was performed on individual schizonts by labeling these with *eba181* and *rh5* specific ViewRNA™ probe sets simultaneously (refer to *Methods*), and transcripts levels were quantified through confocal microscopy imaging. As shown in Fig. 3d, *eba181* transcript intensity shows high variability between individual schizonts, whereas, *rh5*, which is known to be essential for 3D7 invasion^50^, showed considerably lower variability in mRNA levels between individual schizonts. Using a triple labeling protocol, RNA-FISH detected variable degree of transcriptional variability with large variations in the *msp1* signal followed by smaller but still significant fluctuations of *msp3* and *msp7* signal measured in individual schizonts simultaneously (Fig. 3e). Finally, RNA-FISH uncovered considerable cell-to-cell variability of *ama1* mRNA levels (Fig. 3f), which also showed a relatively high VIS (Fig. 3a). This is somewhat contradictory to the “absolute” essentiality of *ama*1, *rh5* and *msp1* for invasion found in previous studies^50–60^ making them one of the top candidates for malaria invasion blocking vaccine. Interestingly, while the RNA-FISH of *ama1, rh5* and *msp1* exhibited significant transcript variability, in no analyzed schizonts this signal was completely abolished. This suggests that even the highly essential genes can exhibit cell-to-cell variation in gene expression; albeit with non-zero low levels that are still sufficient for the parasite invasion. Overall, these results not only provide experimental validation for the HVG status of these genes but also substantiate the MALBAC-based analyses of stochastic gene expression in *P. falciparum* schizonts presented in this study.

### Single cell transcription of host-parasite interaction factors

**Fig. 4:**
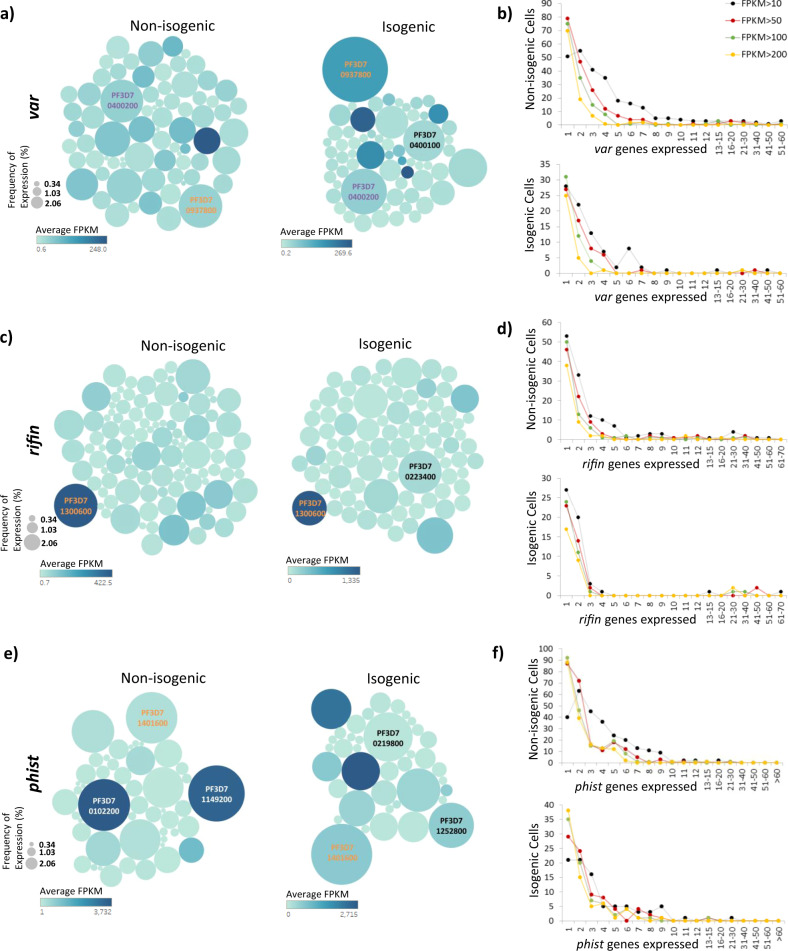
Transcriptional profile of variant surface antigen encoding genes. Bubble plots depicting the frequency of expression and average expression (FPKM) for **a** *var*, **c** *rifin* and **e** *phist* genes in both non-isogenic and isogenic schizonts with same gene IDs highlighted in same colour in the two parasite groups. A distribution of number of **b** *var*, **d** *rifin* and **f** *phist* transcripts expressed per cell is shown as a line plot for non-isogenic and isogenic parasites. Different colours represent the distribution of transcripts expressed above 10 (black), 50 (red), 100 (green) or 200 (yellow) FPKM in individual schizonts. Source data are provided as a Source Data file.

Variant expression of surface exported proteins encoded by gene families such as *var*, *rifin* and *phist* has long been of interest due to their immunogenic, host cell remodeling and cytoadhesive properties, essential for parasite survival during asexual blood stages^15,36,61–63^. As such, understanding cell-to-cell variation patterns of their individual members may shed more light into their biological relevance. Indeed, our results showed that these three families have fundamentally different transcriptional profiles suggesting their distinct roles in host-parasite interactions (Fig. 4a–f). For the *var* gene family, which is believed to be expressed in a mutually exclusive manner, we identify 72 and 62 members out of 105 *var* transcripts (including pseudogenes) expressed at more than 10 FPKM (normalized sequencing reads expressed as fragments per kilobases per million), in the non-isogenic and isogenic parasite populations, respectively. In the non-isogenic schizonts, we found two members, PF3D7_937800 and PF3D7_0400200 detected at the highest frequency in 28.5% and 27.8% of single cells respectively (Fig. 4a). In contrast, the isogenic parasites expressed one dominant *var* gene transcript, PF3D7_937800, at a frequency of 56.7% followed by PF3D7_0400200 and PF3D7_0400100, expressed at 27.8% and 22.7% respectively. This is consistent with the presumed mutual exclusivity suggesting that PF3D7_937800 was the dominant transcript in the “founding cell” of the isogenic population and is still dominant in more than half of the cells. The two other high-frequency *var* genes (PF3D7_0400200 and PF3D7_0400100) likely represent early switches that expanded in the isogenic population within the 14 generations. Even though a single *var* transcript was detected in 17% and 28% of non-isogenic and isogenic cells respectively, a considerable proportion of cells expressed more than one (2–10) *var* transcripts (Fig. 4b). Nonetheless, most of these multiple *var* transcripts are expressed at low levels with a relatively tighter distribution for higher FPKM levels. This suggests that while many cells indeed express one dominant *var* transcript, a significant proportion of parasite cells express additional members. As a comparison, we observed low expression of certain *var* transcripts (~ <50 FPKM) in late asexual stage time points in the bulk 3D7 intraerythrocytic developmental cycle (IDC) reference transcriptome^42^ as well (Supplementary Fig. 11).

In contrast, the *rifin* gene family exhibited a much more restricted expression pattern both in the non-isogenic and isogenic populations. Most *rifin* expressing schizonts had only 1–2 *rifin* transcripts expressed at high levels. This indicates that in schizonts, *rifin* encoding genes are more tightly regulated compared to the *var* genes (Fig. 4c, d). First, 102 and 87 out of 185 *rifin* transcripts (including pseudogenes) were detected at >10 FPKM in at least one of the non-isogenic and isogenic cells, respectively. Out of these PF3D7_1300600 was expressed at the highest frequency of ~12% in non-isogenic parasites. After the isogenic clone expansion, a new *rifin* gene, PF3D7_0223400, gained a slight dominance being expressed in ~14% cells while the original PF3D7_1300600 dropped in its frequency to ~9%. In both groups, the remaining *rifin* transcripts were expressed in less than 11 % of the population with only slight variations between the non-isogenic and isogenic cell populations. Comparatively, we observed low to medium expression of few *rifin* transcripts such as PF3D7_0900500, PF3D7_1300600 and PF3D7_1200500 in bulk 3D7 IDC reference transcriptome^42^.

On the contrary, *phist* encoding genes showed expression in relatively larger proportion of schizonts. Expression of PF3D7_1149200, PF3D7_0102200 and PF3D7_1401600 was most frequent in non-isogenic schizonts at ~45 %, ~38% and ~37% as compared to PF3D7_1401600 (52%), PF3D7_0219800 (34%) and PF3D7_1252800 (28%) in isogenic parasites. Taken together, these data indicate that transcriptional patterns of the *rifin* and *phist* genes undergo only slight shifts upon clonal expansion thus distinguishing these from *var* genes whose mutually exclusive expression is believed to be a key aspect of immune evasion. Interestingly, only a handful of *rifin* genes appear to be expressed in an individual cell, which suggests their redundant biological roles. Conversely, simultaneous expression of a higher proportion of the *phist* gene family may indicate a functional diversification of the individual members most (if not all) of which are required for survival of an individual cell. The ability to change their expression profile after clonal expansion of the parasite population implicate the *rifin* and *phist* gene family in phenotypic plasticity of the malaria parasites as well.

## Discussion

Single cell transcriptomics is a fast-evolving research tool bringing broad and deep understanding of essentially all studied biological systems^13,14,25,64–68^. Without exception, the human malaria parasite has also been a subject of scRNAseq studies in the recent years^26,28–31,69,70^. One of the main achievements was the assembly of the *Plasmodium* cell atlas delineating single cell transcriptomic pattern for essentially all developmental stages of the entire *P. berghei* life cycle, in general^29^. Here, the scRNAseq methodologies were well suited to compare developmental stages to each other and by that to refine differential transcriptional patterns, previously analysed by “bulk” approaches^71–74^. In this study, we wished to complement the previous approaches by focusing on a single developmental stage, to characterize stochastic gene expression as a potential mechanism for parasite phenotypic adaptation. This was done by MALBAC-based WTA that provides an alternative scRNAseq methodology that is more suitable for such studies. In addition to parasite optimised MALBAC presented here, more recently published methodologies, such as, SMARTseq3^75^ and MALBAC-DT^76^ which utilize unique molecular identifiers for mRNA quantification, may be tested as an alternate strategy to study cell-to-cell stochastic variations in future studies.

Using MALBAC based WTA, we studied the schizont stage whose main purpose in the overall *Plasmodium* life cycle is to mature the invasive forms, merozoite, that facilitate parasite proliferation by invading fresh erythrocytes to initiate a new IDC. Here we identify numerous invasion antigens such as *msp1, msp3, msp7, rh5, rhopH2*, *rhopH3, rap1* and *rap3* amongst the HVGs. Our results are consistent with previous studies of transcriptional variation in RBC invasion and merozoite ligands genes in clinical isolates^77,78^. For example, a study on Kenyan patients has shown that distinct clusters of invasion genes are expressed in different isolates^79^ suggesting usage of diverse host erythrocyte receptors by epidemiologically relevant *P. falciparum* strains. Additionally, clonally variant expression of merozoite invasion ligands has been demonstrated before for *eba140, rh4* and *rhopH1* and is thought to be epigenetically regulated^18,80–82^. Upon investigation of the *eba* and *rh* gene families in our dataset, we found two co-expression groups; firstly, *rh2a* (PF3D7_1335400), *rh2b* (PF3D7_1335300), *rh5* along with *msp1, msp3, msp7* and *gap45* transcripts, and, secondly, *rh1* (PF3D7_0402300)*, rh4* (PF3D7_0424200)*, eba140* (PF3D7_1301600)*, eba175* and *eba181* transcripts. This corresponds to both sialic acid (SA) independent and SA dependent invasion pathways being expressed in individual schizonts^83,84^. All these findings indicate that maintenance of variable levels/multiple combinations of invasion transcript repertoire in the population, could afford phenotypic plasticity in scenarios of RBC receptor heterogeneity and ligand-specific immune responses^78,85^. Results in this study suggest that the overall transcriptional variability of the invasion antigens occurs at the cellular level such that not all the invasion antigen are utilized by all cells within a given parasite population. Instead, different (sub)combinations of invasion factors may suffice for a single merozoite to invade a new host erythrocyte.

The observation of the stochasticity of merozoite invasion antigens made by this study could contribute significantly to the overall effort towards the development of an efficacious invasion-blocking vaccine, that up until now focused mostly on single candidate blood-stage vaccines such as MSP1, AMA1, Rh5, EBA-175, MSP2 or MSP3^86–91^. Our results suggest that cell-to-cell variability of expression for most of these antigens might underlay the low efficacies^92–97^ of these strategies reported in the past and that new strategies might require targeting several invasion factors simultaneously. This was previously considered by multi blood-stage antigen vaccine development efforts including the GMZ2 (consisting of conserved domains of GLURP and MSP3) and the MSP1/MSP2 or MSP1/MSP2/RESA triple antigen vaccine^98–100^. As such, multi-target approaches can capture large proportions of invading merozoites helping to overcome additional challenges of invasion blocking vaccines including extensive polymorphism of the surface antigens and the short timespan for which merozoites are exposed to the immune system that requires a very high antibody titre for efficient inhibition^86,87,101^. Indeed, multi-target approach is also considered by the recent alternative strategies utilizing parasite genomic information and reverse vaccinology to discover new blood-stage vaccine candidates^102–105^. Our data on HVGs and stochastic expression of several merozoite surface proteins transcripts can contribute to this by applying a ‘transcriptomics-to-vaccines’ approach. One of such crucial information generated in this study is represented by our discovery of significantly lower cell-to-cell variability of one of the key invasion antigen PfRH5; that contrasts many other antigens (Fig. 3d). Interestingly, PfRH5 is currently considered to have one of the highest potential as invasion vaccine target candidate, whose interaction with erythrocyte surface protein Basigin appears to be invariantly essential for invasion^50,55,56^. Our results strengthen this potential and provide future precedence for cross-referencing of single cell transcriptomics data for new malaria intervention strategies.

The generated high-resolution data set of scRNAseq also allowed us to investigate the set of highly polymorphic gene families, *var, rifin* and *PHIST*, that are also considered by vaccine developmental effort as targetable antigens in infected erythrocytes during *P. falciparum* IDC^106–110^. Specifically, the *pfemp1* (*var*) gene family, whose vaccines are based on a small subset of antigenic types associated with severe malaria^111^ or malaria in pregnant women^106,108^, showed expression of several *var* transcripts expressed at low frequencies across non-isogenic cells with several individual cells showing expression of more than one *var* transcript. This differential transcriptional pattern of *var* genes within a population may possibly contribute (in addition to sequence polymorphism) towards low efficacy of single candidate PfEMP1 vaccines. Furthermore, PfEMP1 variants which are found to be HVG in our dataset, such as, PF3D7_0425800 associated with severe malaria and proposed as an attractive vaccine candidate^111^, must be reconsidered carefully.

Previously, Duffy et al. have demonstrated that multiple *var* transcripts can be detected in single mature trophozoites^112^. Similarly, Chen et al. demonstrated that multiple *var* transcripts can be expressed in single ring-stage infected RBCs, however only one transcript is developmentally regulated to be selected for PfEMP1 expression on the surface of trophozoite infected host cell^113^. Our data reveals for the first time that individual mature schizonts can express up to 10 *var* transcripts. Studies on cell population have demonstrated transcription of both multiple^114^ and single *var* genes^15,115^. Specifically, Taylor et al.^114^. suggest that multiple *var* gene maybe transcribed in a population however only a single full-length *var* transcript remains homogeneous in a population, suggesting possible premature degradation of other *var* transcripts before they can be translated. This could as well be the case in our data, where multiple *var* transcripts maybe present in individual cells contributing towards expression (FPKM) of multiple *var* transcripts possibly indicating potential precursors for *var* gene switching that was estimated to occur in ~2% of each generation^116,117^. Upon cross checking *var* gene expression in bulk 3D7 IDC reference transcriptome, we found expression of several *var* transcripts in schizonts at varying levels albeit lower than the expression in ring stages. The dynamic range of *var* gene expression across life cycle suggests that some of the *var* transcripts are indeed expressed in late stages however these levels are lower (but not necessarily zero) in comparison to ring stages (Supplementary Fig. 11).

In addition to surface antigen encoding genes and invasion genes, we also discovered variation in a wide range of other genes, such as, folate transporter 1, AP2 transcription factor, chromosome assembly factor 1 (PF3D7_0501800), histones, heat shock protein 101 (PF3D7_1116800), *exp2* and calcium-dependent protein kinases involved in key cellular functions, such as, transcription regulation, nucleosome assembly, nutrient/protein channel and parasite egress^118^. This suggests the presence of unique ‘transient’ transcriptional states of individual parasites in a population, some of which may be suitable in defence to antagonists or coping to fluctuating environments; a strategy known as bet hedging. We propose that stochastic expression of diverse gene groups could be a potential mechanism underlying spontaneous adaptation of malaria parasites and future studies in *P. falciparum* parasites under perturbed growth conditions, such as, exposure to drugs, heat shock, nutrient starvation etc. are required to reveal novel mechanisms of phenotypic adaptation in malaria parasites.

Furthermore, in-depth analysis of the generated single cell transcriptomes revealed multiple “layers” of transcriptional variability in the blood stage schizonts of *P. falciparum*. Single schizonts segregated into 36–40 HPI and >44 HPI age groups along the pseudotemporal ordering for both non-isogenic and isogenic group. This is reminiscent of an intrinsic developmental checkpoint during the schizont development (~40 HPI) that is consistent with predicted transcriptional shifts during developmental progression, indicating rapid turning on or shutting down of broad transcriptional modules at once^28^. The existence of an intrinsic developmental checkpoint is also consistent with previous studies demonstrating that nuclear division during schizogony is asynchronous, where individual parasites follow autonomous nuclear division patterns^119,120^. Moreover, variable expression for several crucial biological pathways, such as, protein degradation^121–124^ and protein export (PTEX)^125–127^, which are prime antimalarial drug targets (e.g., proteasome inhibitors), were detected between SCTSs. We propose that our results on DE genes between SCTSs and HVGs can be cross-referenced when exploring future antimalarial drug targets to exclude genes which exhibit high variability (thus making an inefficient target).

In regards to parasite sub-populations, Poran et al. have previously reported segregation of 30, 36 and 42 HPI asexual NF54 *P. falciparum* cells into 11 clusters^69^, however, the authors attributed this segregation to the developmental progression of parasites by correlation to bulk-RNAseq time course. According to our results, isogenic schizonts did not show any sub-structure in the population despite having a similar developmental progression as non-isogenic cells, suggesting that cell-based transcriptional sub-structuring is mediated by cellular differentiation (and not only by cell cycle progression) involving various genetic and epigenetic factors that are known to contribute towards transcriptional (and phenotypic) variation^13,128^. Better understanding of the mechanisms underlying cellular divergence will undoubtedly improve our knowledge of malaria parasite phenotypic plasticity, which could aid considerably in the development of future malaria intervention strategies involving all drugs, vaccines and diagnostics.

In addition to stochastic gene expression, our results uncovered a broad spectrum of SNPs that appear to rise in individual cells in otherwise genetically coherent parasite populations. Curiously, in the isogenic parasites that were generated by a clonal expansion over 14 generations, we identified 1,185 exonic SNPs. This is apparently higher than the expected number of basal-level genomic SNPs in *P. falciparum*^129,130^, however, this is likely due to the fact that in this study we focused on the schizont specific genes that are overrepresented by factors of host-parasite interactions that are known to exhibit higher genetic variability^130^. Indeed, visual inspection of the SNPs identified in the isogenic parasites show their skewed distribution in multiple members of gene families involved in merozoite invasion including merozoite surface antigens (MSP), reticulocyte binding protein homologues (RH), cytoadherence-linked asexual proteins (CLAG) that are also known to be highly variable (Supplementary Data 8). A recently published single cell genomics study of *P. falciparum* parasites similarly revealed higher concentration of genetic variations in certain gene families such as *msp3* (1 SNP/12 bp) and *sera* (1 SNP/ 23 bp)^131^. Additionally, RNA-specific mutations that are known to be introduced erroneously during the transcription process in all eukaryotic systems cannot be ruled out and thus might account for some of the detected polymorphism in addition to the background genomic SNPs. Furthermore, isogenic cells were derived after short-term culturing for 28 days (14 generations) as compared to clonal parasite lines being cultured for long term ranging from ~100 to 1060 days for SNP identification in previous studies^129,130^. This could result in the capturing and detection of all the newly arisen early SNPs which haven’t yet been selected against and “diluted out” or eliminated from the culture. Overall, it is feasible to speculate that the increased number of SNPs in schizont specific genes goes hand in hand with the stochastic gene expression, both of which reflect the main focus of this developmental stage; the production of invasive merozoites for re-invasion of fresh host erythrocytes while evading the immune response when exposed to the blood plasma factors.

## Methods

### Experimental model

*P. falciparum* 3D7 strain was maintained in RPMI 1640 medium (Gibco) supplemented with Albumax I (Gibco) (0.25%), hypoxanthine (Sigma) (0.1 mM), Sodium bicarbonate (Sigma) (2 g/L), and gentamicin (Gibco) (50 μg/L) in 2% haematocrit at 37^o^C on shaker. Cultures were gassed with malaria gas (5% CO_2_, 3% O_2_, and 92% N_2_) after daily medium change. Freshly washed human RBCs were added to the culture every alternate day. Parasitemia and parasite morphology were assessed daily by microscopic examination of blood smears stained with Giemsa (1:10 dilution, Sigma). For synchronisation, ring stage culture were treated with 5% D-Sorbitol for 15 min with intermittent shaking. Treated culture were washed twice in culture media (2000 rpm, acc 9, brake 1) and resuspended in fresh medium.

### Single cell sorting

A sorbitol synchronised *P. falciparum* 3D7 schizont culture (40 to 44 HPI) was used for performing single cell sorting. 20 µl of infected RBCs (iRBCs) were washed in 1X PBS twice and stained with SYBR green dye (final dilution 0.2 x) for 30 min at 37 ^o^C in the dark. Next, the iRBCs pellet was washed >5 times with 1X PBS before final resuspension in 1X PBS solution ready for FACS. Uninfected RBCs were also stained in parallel as a control for gating strategy (Supplementary Fig. 15). iRBCs were sorted on BD FACSAria™ into 4 µl of lysis buffer (5x FS buffer = 100 µl, 0.1 M DTT = 25 µl, 10 mM dNTP= 25 µl, 40 U/ul RNaseOUT Inhibitor™ = 25 µl, GAT-dT Primer (100 µM) = 12.5 µl, H2O = 311.5 µl, Triton X114 = 1 µl) in 96 well plates, spinned briefly and processed immediately or stored at −80 ^o^C for later. BD FACSDiva™ software (8.0.1) was used for analysis.

### MALBAC whole transcriptome amplification

Single cell lysate obtained after FACS was subjected to reverse transcription and WTA using MALBAC protocol optimised for *P. falciparum* transcriptome. Briefly, single cells were collected in 4 µl of lysis buffer prepared as follows; 100 µl of 5X First Srand Buffer (Invitrogen), 25 µl of 0.1 M DTT, 25 µl of 10 mM dNTP, 25 µl of 40 U/L RNaseOUT Inhibitor™, 12.5 µl of 100 µM GAT-dT primer, 311.5 µl of nuclease free water and 1 µl of Triton X-114. Subsequently, cell lysate was exposed to 70 degrees for 90 s in a thermocycler. For reverse transcription 0.33 µl of SuperScript II, 0.07 µl of T4 gene32 and 0.05 µl of RNaseOUT inhibitor™ were added to the lysate and thermocycled at 4 °C, 2 min, 10 °C, 2 min, 20 °C, 2 min, 30 °C, 2 min, 40 °C, 2 min, 42 °C, 50 min, 70 °C, 15 min and finally maintained at 4 °C, hold. Next, first strand cDNA was amplified by performing 35 cycles of MALBAC (at 95 °C 5 min, and then 35 cycles of 20 °C 50 s, 30 °C 50 s, 40 °C 45 s, 50 °C 45 s, 65 °C 4 min, 95 °C 20 s, 58 °C 20 s) by adding 0.15 µl of 100 uM GAT-7N primer, 1.6 µl of 10 X Thermo Buffer, 0.64 µl of 10 mM dNTP, 0.16 µl of 100 mM MgSO4, 0.48 µl of 2U/ul *Pyrococcus* derived Deep Vent® DNA polymerase and 7.97 µl of nuclease-free water. This was followed by 19 cycles of PCR set up as 0.15 µl of 100 uM GAT-COM primer (final concentration 0.5 µM), 0.56 µl of 10 mM dNTP, 1.4 µl of 10X Thermo Buffer, 0.14 µl of 100 mM MgSO4, 0.42 µl of 2U/ul *Pyrococcus* derived Deep Vent^®^ DNA polymerase and 11.43 µl nuclease free water added to the MALBAC product generated in the previous step. PCR cycling was performed at 95 °C for 1 min, 19 cycles of 95 °C 20 s, 58 °C 30 s, 72 °C 3 min, and a final 5 min additional extension at 72 °C. Primer sequences for each step are: GAT-12dT. 5'- GTG AGT GAT GGT TGA GGT AGT GTG GAG TTT TTT TTT TTT −3' (used during reverse transcription); GAT-7N. 5'- GTG AGT GAT GGT TGA GGT AGT GTG GAG NNN NNN N −3' (used during MALBAC amplification); GAT-COM. 5'- GTG AGT GAT GGT TGA GGT AGT GTG GAG −3' (used during PCR). Final amplified cDNA was purified using Agentcourt Ampure XP magnetic beads according to the manufacturer’s instructions. All non-isogenic cells were amplified in five batches whereas isogenic cells were processed in a single batch.

### SMARTseq2 amplification

For SMARTseq2 based WTA, 1-cells were processed according to the Picelli et al. protocol^41^ with modifications. Briefly, 1-cells were sorted in 3 µl of lysis buffer (bovine serum albumin 1 mg/ml, 2U/µl RNaseOUT Inhibitor™). Lysate was spinned down briefly and stored at −80 °C until further use. RNA-primer hybridization and reverse transcription was performed following Picelli et al.^41^. Preamplification was performed using entire first strand cDNA, KapaHIFI 2x PCR mix, 0.5 µM ISPCR primer and 3 mM MgCl_2_ with annealing and extension at 56 °C and 64 °C respectively for 25 cycles. Final amplified cDNA was purified using Agentcourt Ampure XP magnetic beads according to the manufacturer’s instructions.

### RNA sequencing

Purified MALBAC amplicon was used directly for preparing sequencing libraries using Illumina Nextera XT kit following manufacturer’s instructions. Purified cDNA libraries were QC’ed on Agilent Bioanalyser 2100 using High-Sensitivity DNA chips. Libraries with optimal size distribution of 300 to 900 bp were pooled (20 samples per lane) and sequenced on Illumina HiSeq4000 platform generating 150 b x 150 b paired end reads with 110 Gb data output generated per lane. A minimum of 25 million reads were generated per library. Non-isogenic cells were sequenced in five batches whereas isogenic cells were sequenced in a single batch. All SMARTseq2 and MALBAC amplicons for optimization were sequenced on HiSeq2500 platform (Supplementary Fig. 1) and Novaseq platform (Fig. 1).

### Sample genotyping and SNP analysis

Polymorphism discovery was performed using RNA short sequence reads following the process applied to DNA reads that was described elsewhere^132^. In brief, we aligned the raw reads from an individual sample of isogenic or non-isogenic cells against the *P. falciparum* 3D7 reference sequence V3 using the Hisat2 program as above described. As recommended by GATK best practices aligned reads covering the splice junctions split into exon specific sequence by SpliNCigarReads. Base qualities were recalibrated using Pf3K known sites. GATK HaplotypeCaller was used for the joint genotyping. To achieve a high-quality set of SNPs, we set stringent filtering criteria: 1) Each polymorphism needed to be covered by more than 10 reads (Supplementary Fig. 7b) with GATK quality score (GQ) greater than 30 in >20 cells (isogenic/non-isogenic). 2) To reduce the complexity of the analysis, we only considered biallelic SNPs within coding regions. To this end, there were 3459 SNPs that passed the threshold with the alternative allele present in at least 1 cell (isogenic/non-isogenic). Out of the 3459 SNPs, 1185 are detected with polymorphisms only in isogenic cells and 2224 only in non-isogenic cells. For the 50 SNPs (1.4% of the 3459 SNPs) showing polymorphisms in both, majority occurred with low minor allele counts as 39 SNPs (78%) has the minor allele counts less than 4 and 45 SNPs (90%) less than 6. Only 284 SNPs showing 50% representation in isogenic or non-isogenic schizonts was used for population genetic structure analysis (Supplementary Figs. 7 and 14).

### qRT-PCR

qRT-PCR was performed on purified MALBAC amplicon from individual schizonts for targeted genes using the Applied Biosystems™ SYBR™ Select Master Mix. Forward and reverse primers for each gene were designed using the NCBI Primer BLAST tool with annealing temperature set to 60 °C and product size limited to 150 to 250 bp. All primers were ordered from Integrated DNA Technology at 100 µM stock concentration. A cDNA input of 5 ng was used per sample during qRT-PCR for normalised comparison of individual transcript level (represented by Ct values) variation between single cells. z-score for each gene was calculated by the following formula: *z* = (C_tcell_-µC_tPfRNAdil_)/δC_tPfRNAdil_

### RNA-Fish and imaging

Customised RNA-FISH probes for individual genes were ordered from Thermo Fisher Scientific. Prior to performing RNA-FISH, thin smears of *P. falciparum* 3D7 schizont culture (38 to 42 hpi) was made on polylysine coated glass slides. Upon air drying, smears were fixed using 4% PFA and 0.008% Glutaraldehyde solution for 45 min. The rest of the steps of RNA-FISH labelling was performed using the Invitrogen^™^ ViewRNA^™^ ISH Cell assay kit following manufacturer’s instructions. Stained blood smears were imaged using confocal microscope LSM710 at 500× (optical zooming) and image analysis was performed on ZEN 3.0 (blue edition ZEN lite) software.

### Subcloning

*P. falciparum* 3D7 parasites were subcloned using serial dilution cloning. Culture was diluted in uninfected RBCs suspended at 2% haematocrit in culture media until 0.1 to 0.5 parasites were obtained per well theoretically. Media was changed on day 4, day 7, day 10 and every other day thereafter. 1 to 2 µl of fresh RBC were added to each well from day 7 onwards on every third day. Positive wells were screened from day 14 onwards by Hoechst staining (8 mM) on BD FACSAria^™^ (version 6.0).

### Data analysis

Raw reads obtained from the sequencer were checked for overall quality and trimmed to remove low quality bases from 3’ ends, adapters, and amplification primers using Trim Galore (version 0.6.6). Fastq-pair was used for read processing and Samtools was used for alignment processing. HISAT2 aligner was used to perform alignment to the *P. falciparum* genome. Only paired reads with proper orientation mapped to unique location of the genome were considered for counting. Gene specific read counts were calculated using BEDTools. Fragments per kilo base per million mapped reads (FPKM) were then calculated and used for further analysis.

### Single-cells clustering

To investigate the transcriptomic heterogeneity of the parent parasite population, we applied clustering analysis to the 295 single cells. Dropout rate, batch effect, over-dispersion and the count nature of the data was accounted for using the zero-inflated negative binomial model (ZINB-WaVE)^43^. Before clustering, we filtered out those samples having a significantly lower (3 time mad lower than median) mapping rate or total read counts; also samples were discarded if they presented <75% of the 269 highly presented genes which were detected in 95% of PfRNAdil samples. Finally, there were 271 samples used for further study. Here, we considered a total of 4934 genes which were detectable in at least 5 cells excluding mitochondrial genes. The 271 single-cells were clustered using the method of RSEC implemented in R with the package ClusterExperiment^133^. The low-dimensional matrix used for clustering was specifically computed using the zinbwave function of the R package zinbwave where K = 50 was set for dimensionality reduction. Sample processing batch information was used as one of the covariates during zinbwave analysis to remove any batch effects. In practice, we applied k-means to generate a collection of candidate clustering using k0s = 4:5; obtained the consensus clustering based on the setting consensusProportion = 0.7, consensusMinSize = 5 and minSizes = 1; sequentially merged similar clusters based on the setting alphas = c(0.2), betas = 0.75, clusterFunction = “hierarchical01” and seqArgs =  list(remain*.n* = 10, top.can = 5). And the stability of clusters were controlled by 500 times subsampling with 70% samples used each time as the parameters were set as subsampleArgs = list(resamp.num = 500, samp.*p* = 0.7, clusterFunction = “kmeans”, clusterArgs = list(nstart = 5)). Then, we asked the RSEC to merge the resulted clusters which had less than 15% differential expression using mergeMethod = “adjP” and mergeCutoff = 0.15 (Supplementary Fig. 12a). Lower mergeCutoff at 0.05 only generated smaller clusters within two of the clusters from mergeCutoff = 0.15 setting (Supplementary Fig. 12b). At last, we obtained 8 major clusters with 196 of 271 samples. We assigned the left 75 samples, which form clusters with less than 5 cells under the required conditions, to their nearest cluster using the assign unassigned function of ClusterExperiment. We repeated the same procedure of clustering analysis for isogenic cells. Out of 97 single-cells from a isogenic clone, 79 passed the filtering criteria and their transcriptome were used for the cluster detection by RSEC algorithm. Given the smaller number of single-cells studied here, we reset the parameters of consensusMinSize = 5 and seqArgs = list(remain.*n* = 10, top.can = 3). At last, a single cluster was obtained at the same criteria above that >10% differential expression were required to create two distinct clusters. We also lowered down the mergeCutoff to 0.05 which reported 5 clusters as shown in Supplementary Fig. 13 with maximum 8% differential expression between clusters. To account for sampling size difference between non-isogenic and isogenic schizonts, we downsized the non-isogenic data to 80 samples to match the number of isogenic samples by sub-sampling algorithm and re-applied the RSEC method using 428 highly representative genes. The high representation was defined as genes present in all the fifteen samples of 100-cells, >50% PfRNAdil samples and >5% 1-cell samples. The resulted 682 genes were further pruned to 428 genes as we asked for greater SD across single cells than PfRNAdil. The sub-sampling was repeated 50 times with the RSEC analyses, and the result converged after 20 times. For isogenic schizonts, clusters disappeared at a cluster merging cutoff of <25% differential gene expression (DE) between clusters (Supplementary Fig. 5a). And the multiple SCTS in non-isogenic schizonts was maintained under the same criteria in majority of the times (62% confidence, Supplementary Fig. 5b).

### Reporting summary

Further information on research design is available in the Nature Research Reporting Summary linked to this article.

## Supplementary information

Supplemementary Information

Description of Additional Supplementary Information

Supplementary Data 1

Supplementary Data 2

Supplementary Data 3

Supplementary Data 4

Supplementary Data 5

Supplementary Data 6

Supplementary Data 7

Supplementary Data 8

Reporting Summary

## Data Availability

Full RNA sequencing dataset generated during this study are available on GEO database with accession number GSE164459. The 3D7 reference transcriptome data can be accessed through GEO accession number GSE150484. Source data are provided with this paper.

## Source data

Source Data

## Code availability

The code generated during this study has not been deposited in a public repository because most tools used for analysis are publicly available. The quality trimming code is available from the corresponding author upon request.
