# Supplementary material for: Stochastic expression of invasion genes in Plasmodium falciparum schizonts
Source: Nat Commun. 2022 May 30;13:3004. doi: 10.1038/s41467-022-30605-z (PMC9151791; doi:10.1038/s41467-022-30605-z)
Supplement: Supplementary file 1 — Supplemementary Information [file 41467_2022_30605_MOESM1_ESM.pdf]

**Supplementary Information**

# Stochastic Expression of Invasion Genes in *Plasmodium falciparum* Schizonts

Jaishree Tripathi[1]*, Lei Zhu[1], Sourav Nayak[1], Michal Stoklasa[1], Zbynek Bozdech[1,]*

[1]School of Biological Sciences, Nanyang Technological University, Singapore, 637551

*Corresponding authors: tjaishree@ntu.edu.sg , zbozdech@ntu.edu.sg

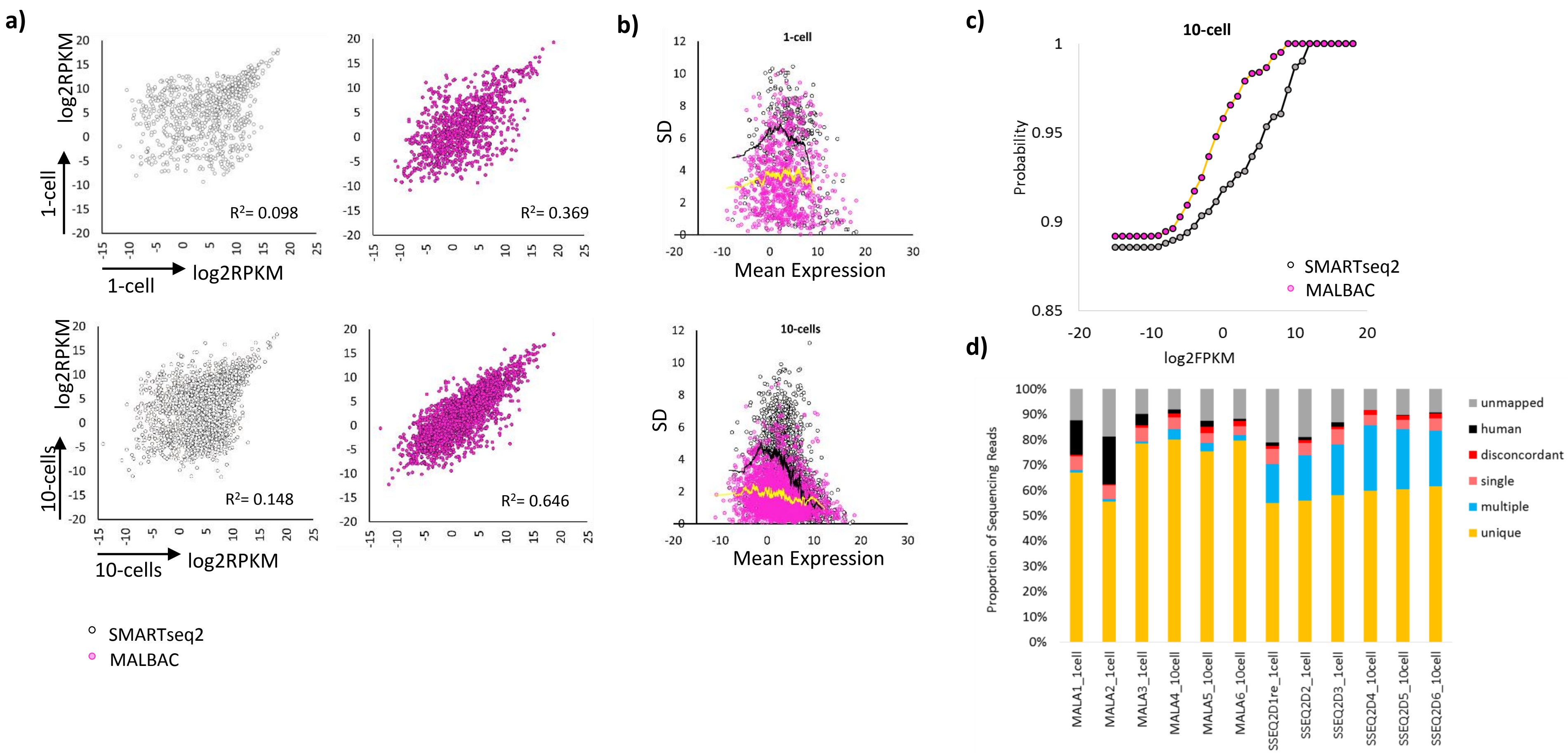


**Supplementary Figure 1 (Related to Figure 1)**: **Sequencing Read Alignment Summary for Various Whole Transcriptome Amplification Methods Tested**. a) Scatter plots showing correlations between 1-cell and 10-cells transcriptomes amplified by SMARTseq2 (grey) or MALBAC (magenta). Transcript expression is shown as log2RPKM values on the x and y-axis. b) A scatter plot showing relationship between standard deviation (SD) and mean transcript expression (log2RPKM) across 1-cell and 10-cells triplicates for the two techniques. c) A scatter plot showing the probability of detection of transcripts across 10-cells triplicates amplified by MALBAC or SMARTseq2. d) A bar graph representing the proportion of sequencing reads that mapped to the *P. falciparum* genome either uniquely, at multiple loci, discordantly, singly, unmapped or mapped to the human genome after whole transcriptome amplification of 1-schizonts and 10-schizonts triplicates using either modified MALBAC or modified SMARTseq2 method. All samples were sequenced on HiSeq2500 platform.

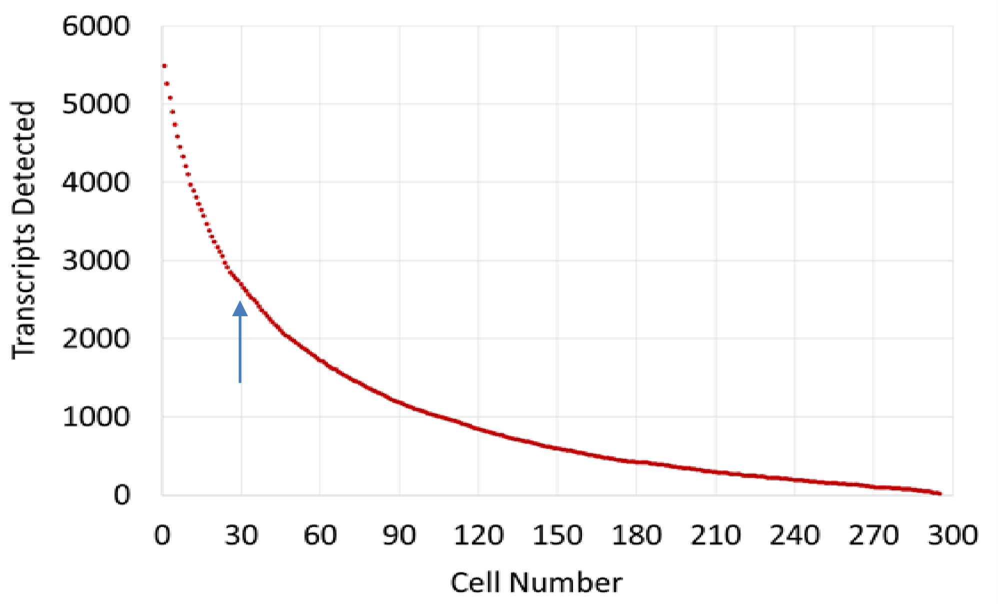


**Supplementary Figure 2 (Related to Figure 1)**: **Cumulative Transcript Coverage in Schizont Population.** A dot plot depicting the cumulative number of transcripts detected in the 295 schizonts sequenced from non-isogenic *P. falciparum* 3D7MR4. Blue arrow represents the number of transcripts expressed in more than 10 % of cells.

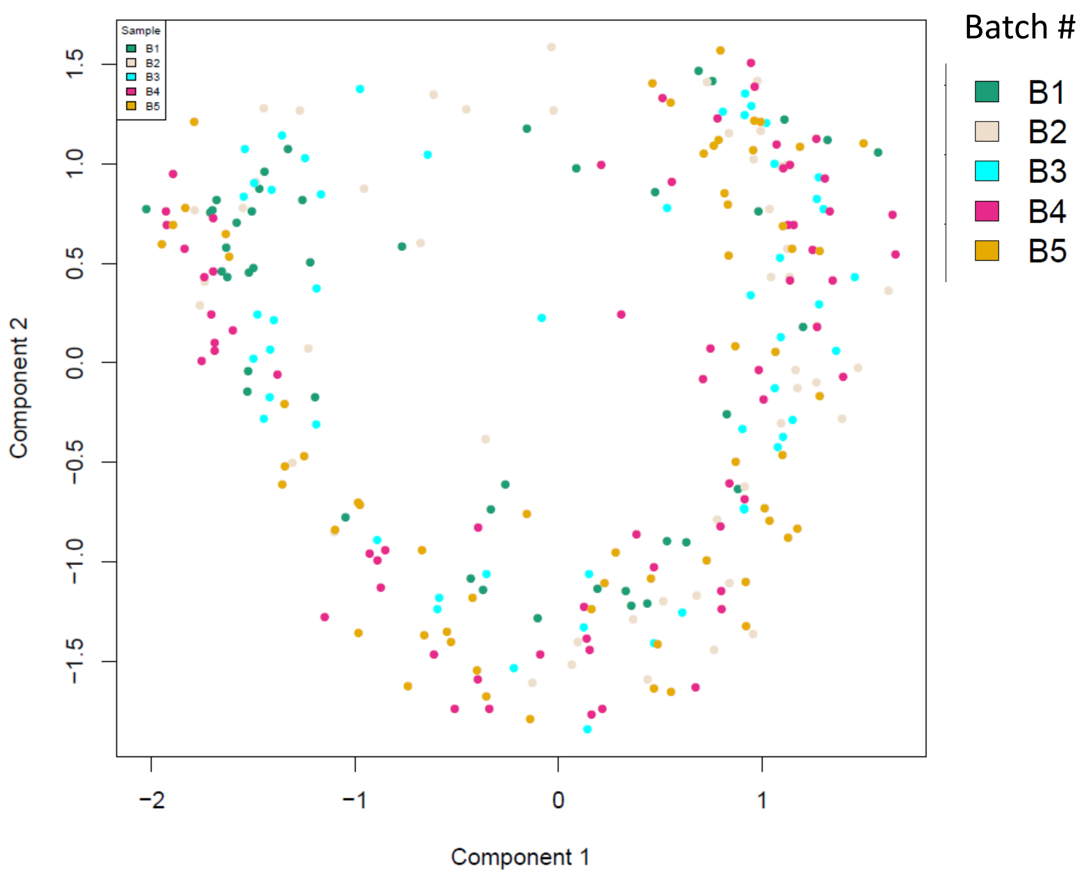


**Supplementary Figure 3:** PCA distribution of non-isogenic cells after batch effect removal during dimensionality reduction analysis (ZINB-WaVE ).

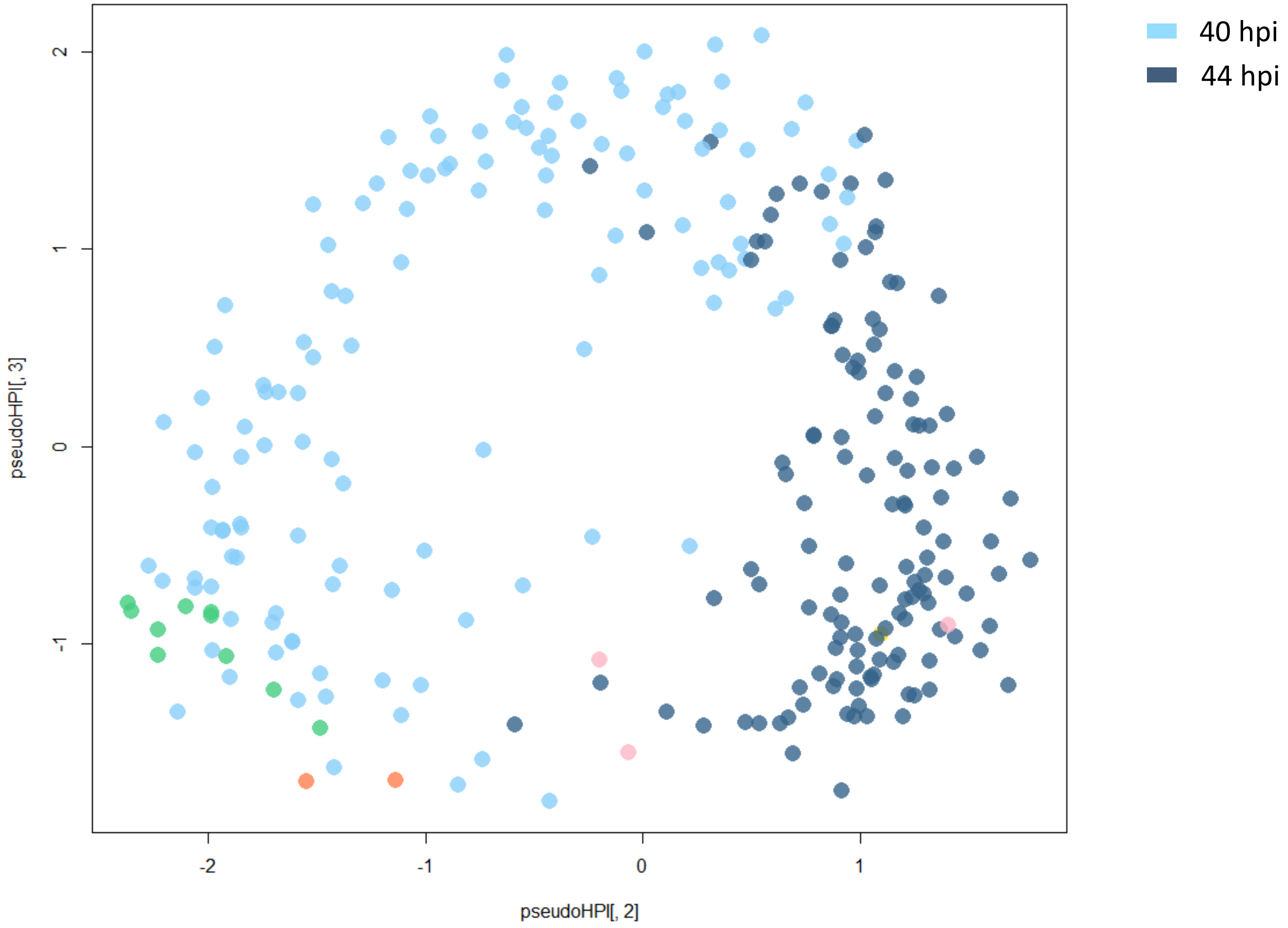


**Supplementary Figure 4:** Age estimation projected upon pseudotime trajectory of non-isogenic cells showing majority of cells at 40 hpi and 44 hpi.

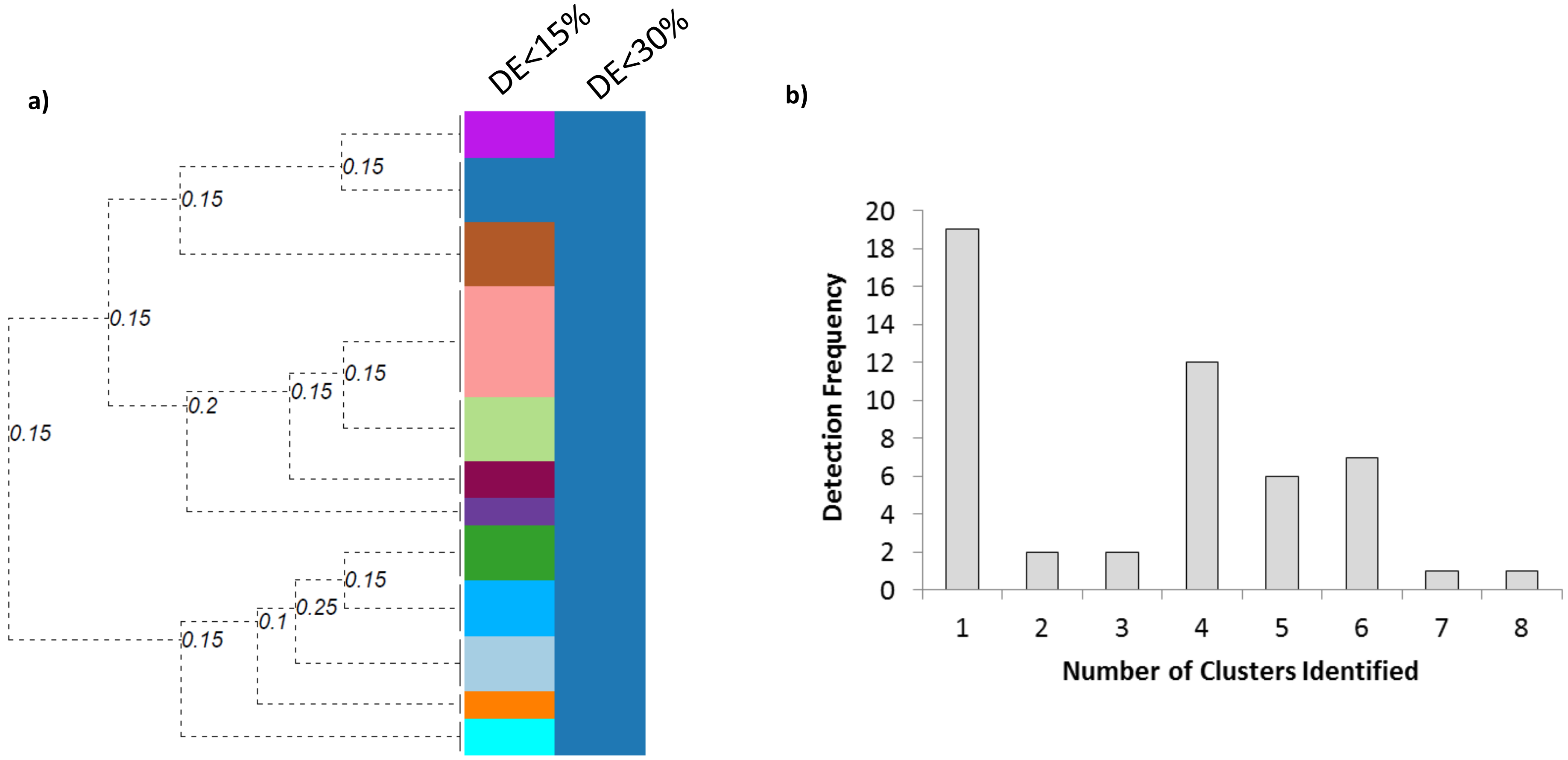


**Supplementary Figure 5:** a) RSEC clustering for isogenic schizonts shows SCTS sub-populations merging at a differential gene expression cut off of >25 % between clusters. b) Number of clusters identified upon sub-sampling of 80 single cells randomly from the 271 non-isogenic schizonts.

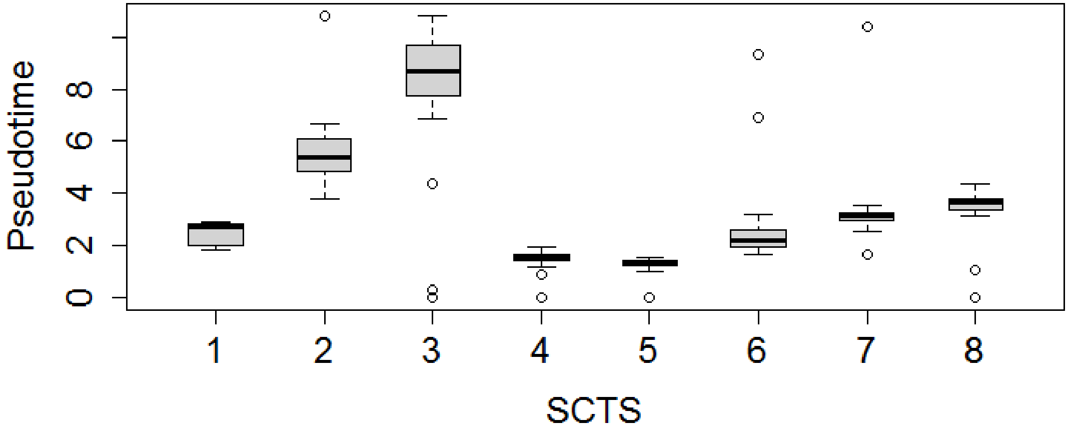


**Supplementary Figure 6:** Boxplots showing distribution of pseudotime values for each SCTS detected in non-isogenic schizonts (n=271 1-schizont). For each boxplot the centre line depicts the median, box limits as upper and lower quartiles, whiskers as minimum to maximum values.

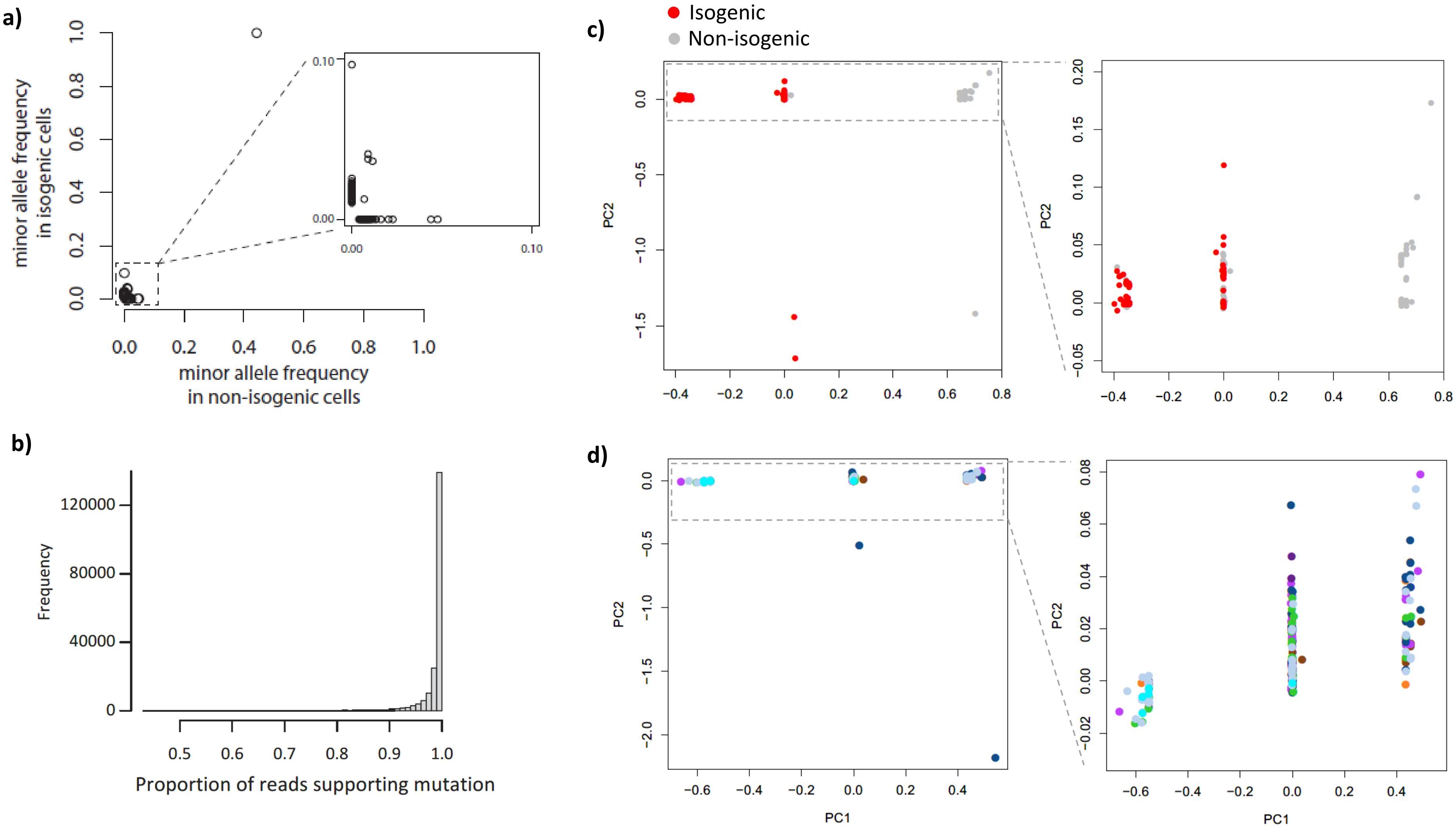


**Supplementary Figure 7:** a) Scatter plot showing minor allele frequency for 284 SNPs in isogenic and non-isogenic schizonts. b) The histogram shows the proportion of reads supporting the genotype at 198140 sites (out of 937389=3459 SNPs * 271 cells) which have at least 10 unique reads covered. c) A PCA plot showing the genetic structure of the isogenic and non-isogenic cells based on the 284 SNPs represented in 50% non-isogenic or 50% isogenic cells. The isogenic cells (red) overlaid to only sub groups of the non-isogenic cells (grey). The segmentation within isogenic cells can be due to absent detection of SNPs. d) A PCA plot of non-isogenic cells based on 284 SNPs showing cells coloured by SCTS.

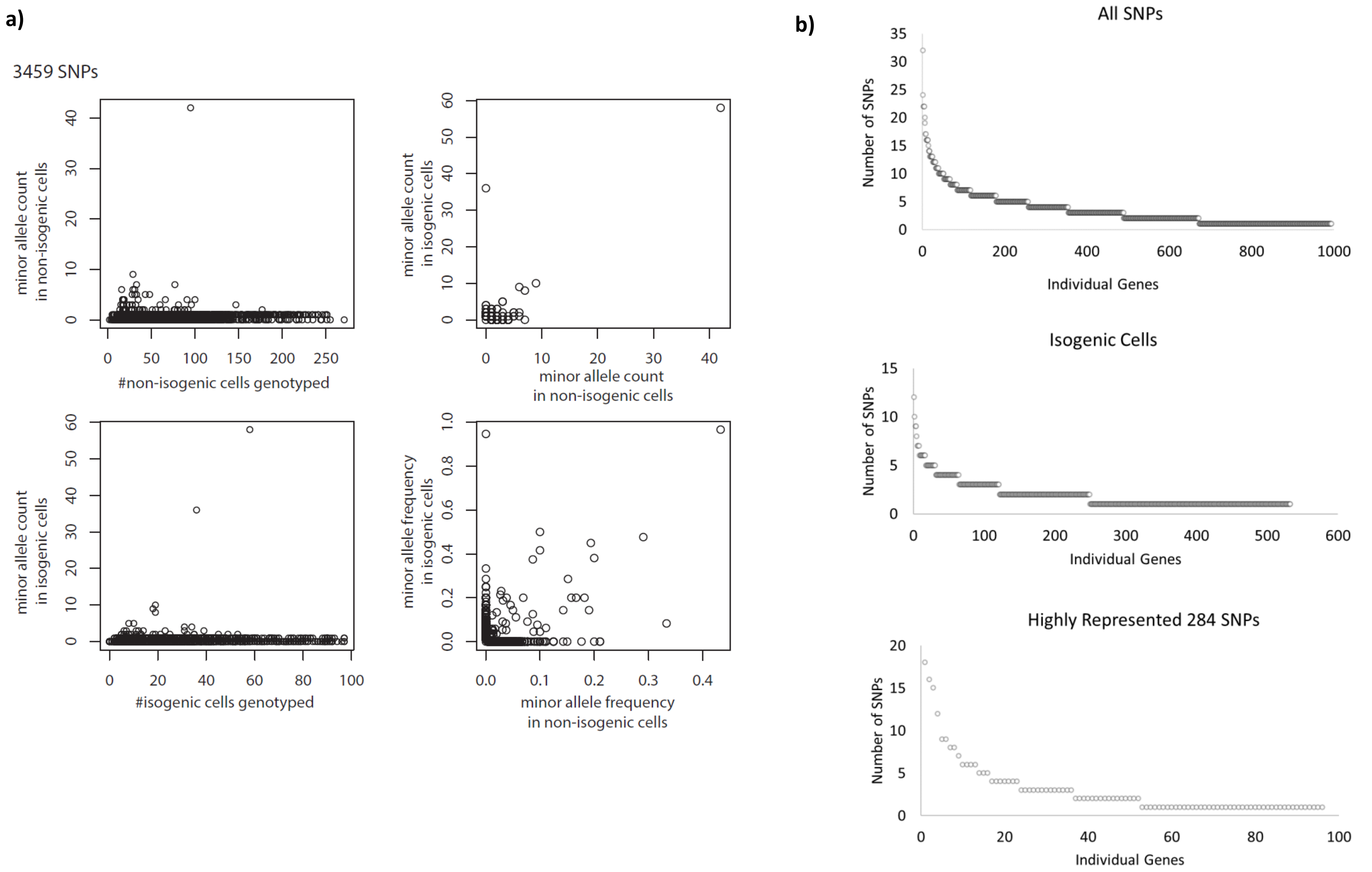


**Supplementary Figure 8:** a) Scatter plots depicting minor allele counts and minor allele frequency in non isogenic and isogenic cells for the 3459 SNPs. b) Scatterplot showing SNPs distribution in individual genes for all 3459 SNPs, SNPs detected in isogenic cells and 284 highly represented SNPs.

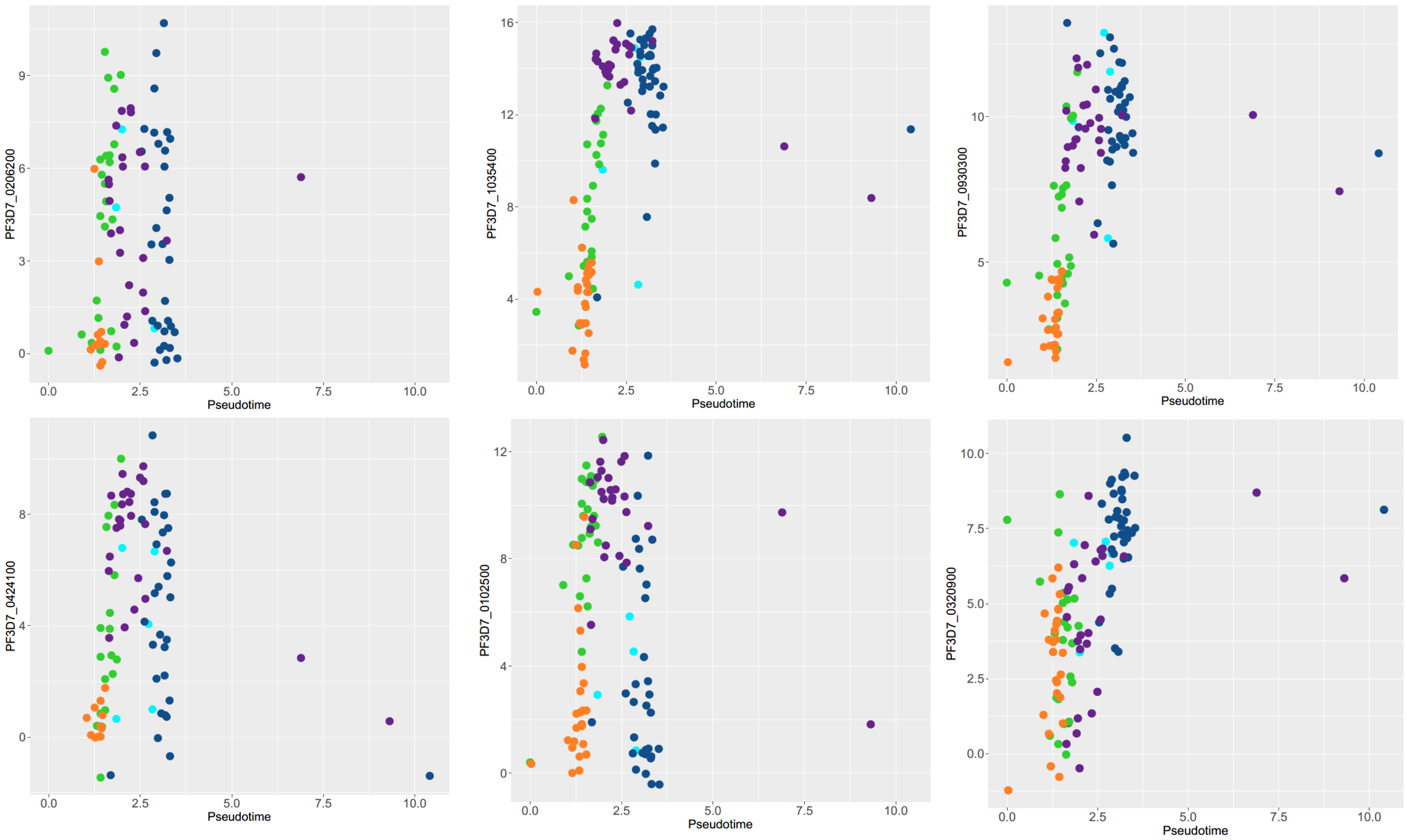


**Supplementary Figure 9:** Scatterplots showing expression of individual HV genes expression along pseudotime. Each dot represents individual schizonts coloured by SCTS.

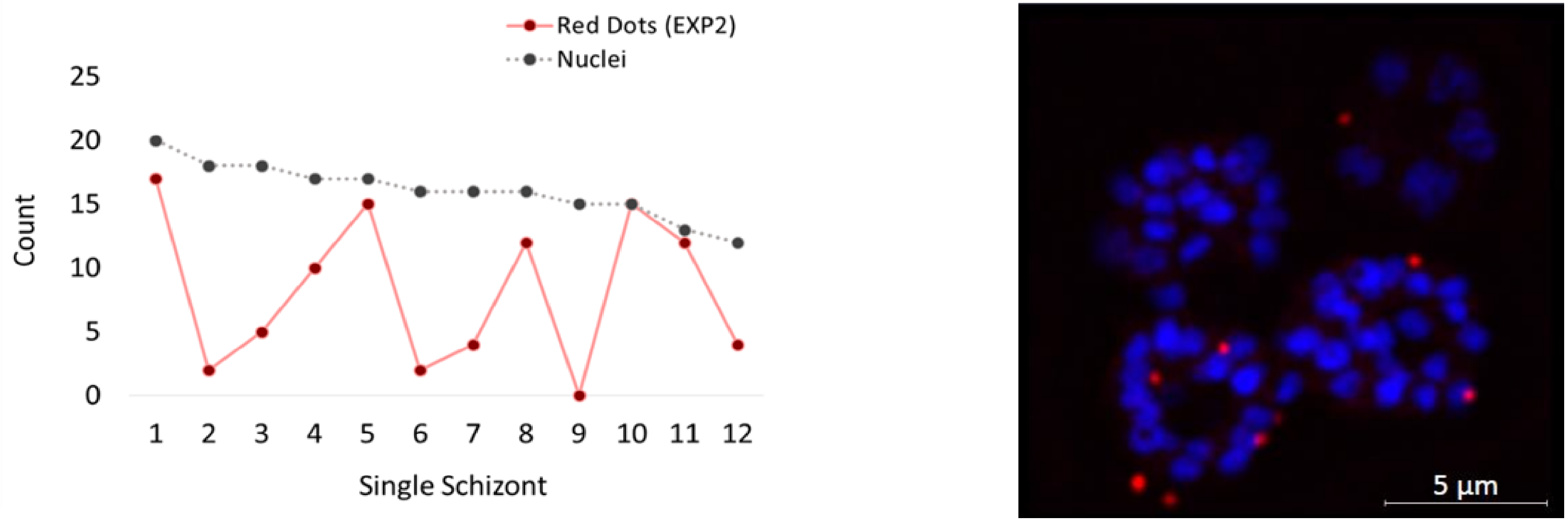


**Supplementary Figure 10 : Variable Expression of EXP2 Transcript Confirmed by RNA-FISH in 3D7MR4 Schizonts.** A line graph showing the number of EXP2 mRNA probe dots observed in individual 3D7MR4 schizonts imaged by confocal microscopy. Parasite nuclei was stained with DAPI. Scale bar = 5 µm.

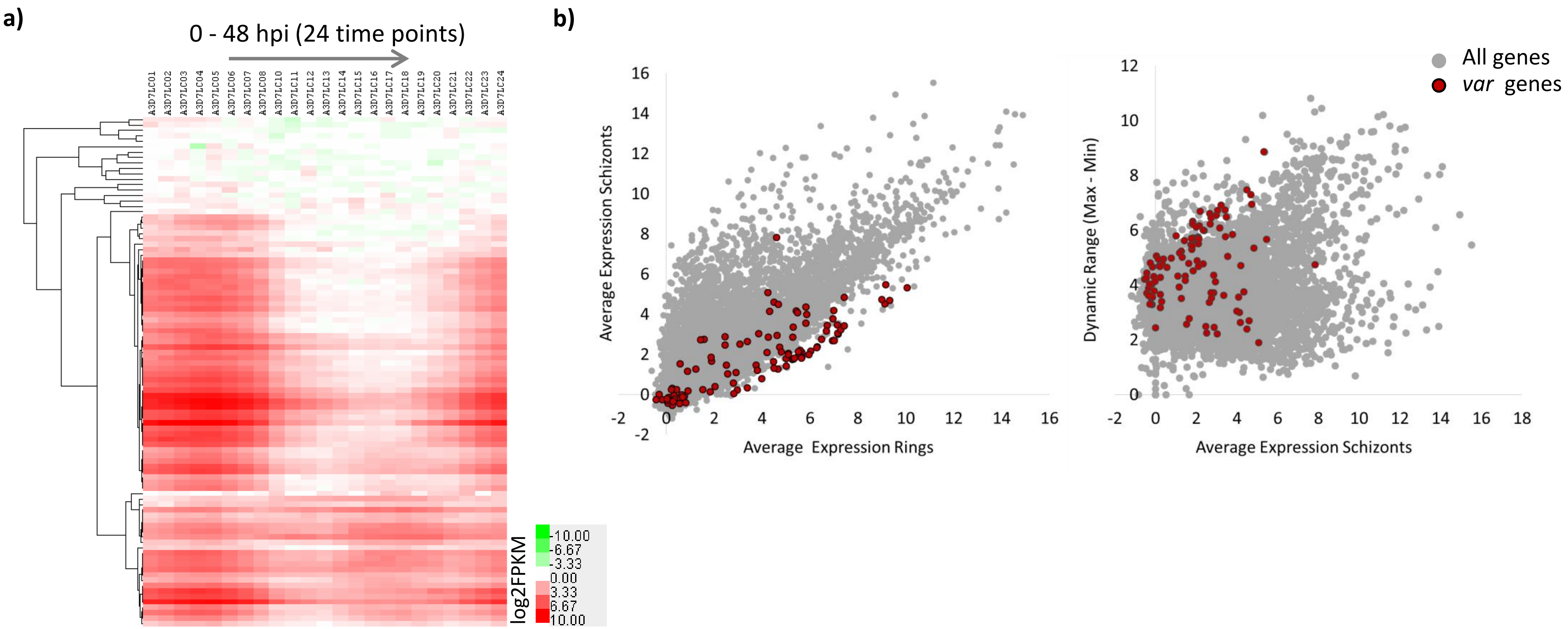


**Supplementary Figure 11:** a) A heatmap showing expression of *var* genes across 3D7 lifecycle (0 to 48 hpi). b) Scatterplots showing average expression of *var* genes in ring stages versus schizont stages (left panel) and the dynamic range of expression of *var* genes across the whole life cycle in relation to average expression in schizonts (right panel).

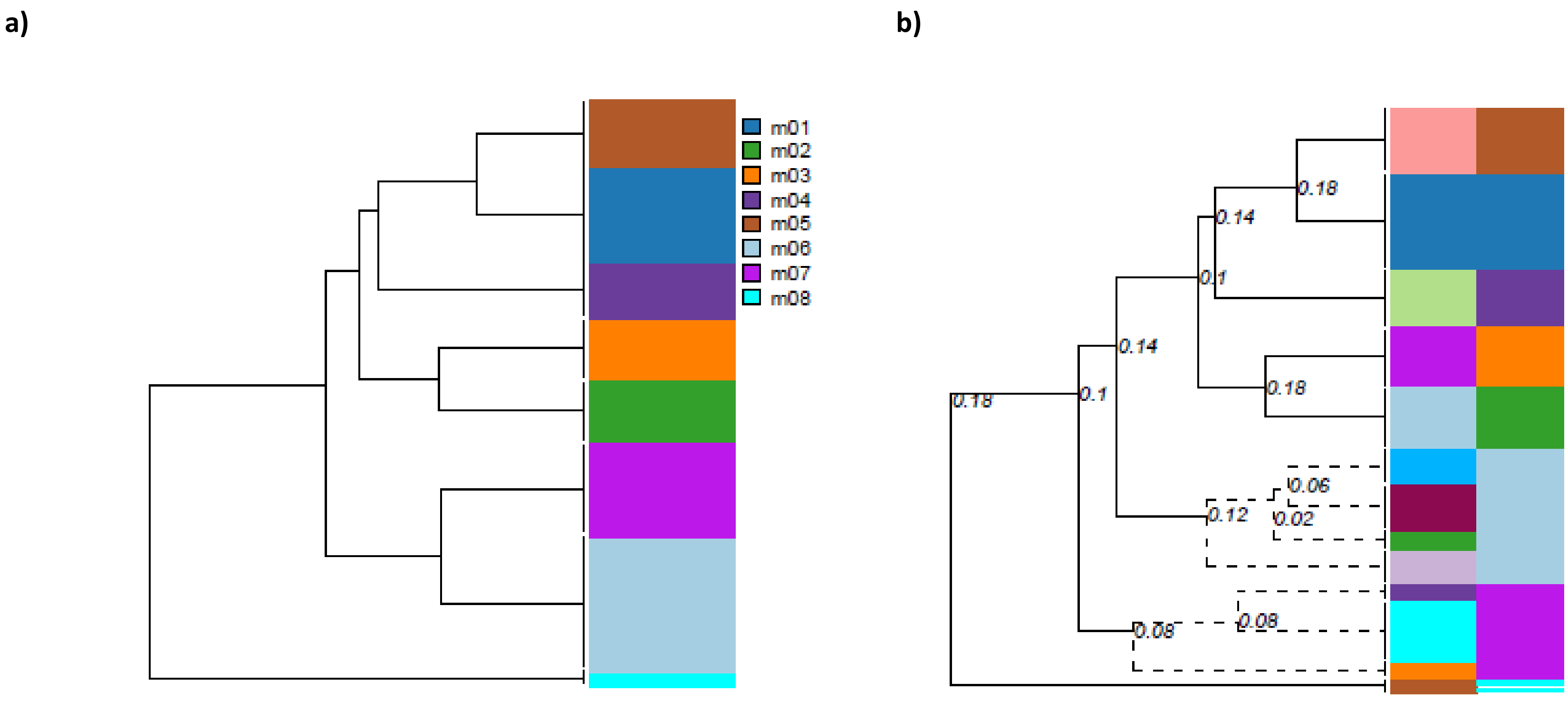


**Supplementary Figure 12 (Related to 'Single Cell Clustering' in *Methods*):** RSEC Based Clustering of Non-isogenic Schizonts. (a)The dendrogram represents the RSEC constructed 1-cell clusters (m01 to m08) with the merging criteria of <15% differential expression between clusters. (b) The dendrogram and blocks represent the merging steps for clusters constructed at lower threshold (blocks on the left column) compared to clusters constructed at higher threshold (blocks on the right column, same as clusters shown in **a**). The dotted lines indicate the merging steps from clusters in **b** to clusters in **a**. Here, the lower merging threshold was set as 5% differential expression between clusters with only cluster m06 and m07 showing further segregation at this lower threshold. This suggests the robustness of the clustering result at the merging threshold of 15%. The numbers along the dendogram indicate differentially expressed genes between the various parasite clusters.

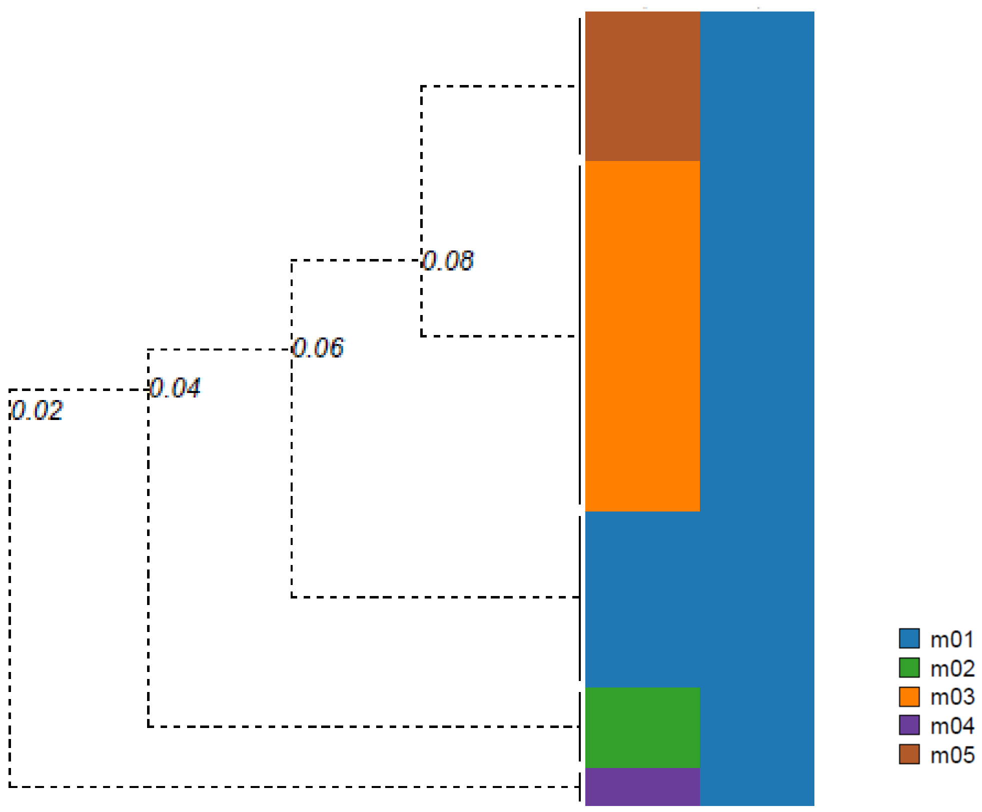


**Supplementary Figure 13 (Related to 'Single Cell Clustering' in *Methods*) :** RSEC Based Clustering of Isogenic Schizonts. The dendrogram and blocks represents the merging process of clusters formed at threshold of 5% (blocks on the left) to 15% (blocks on the right) differential expression between parasite clusters.

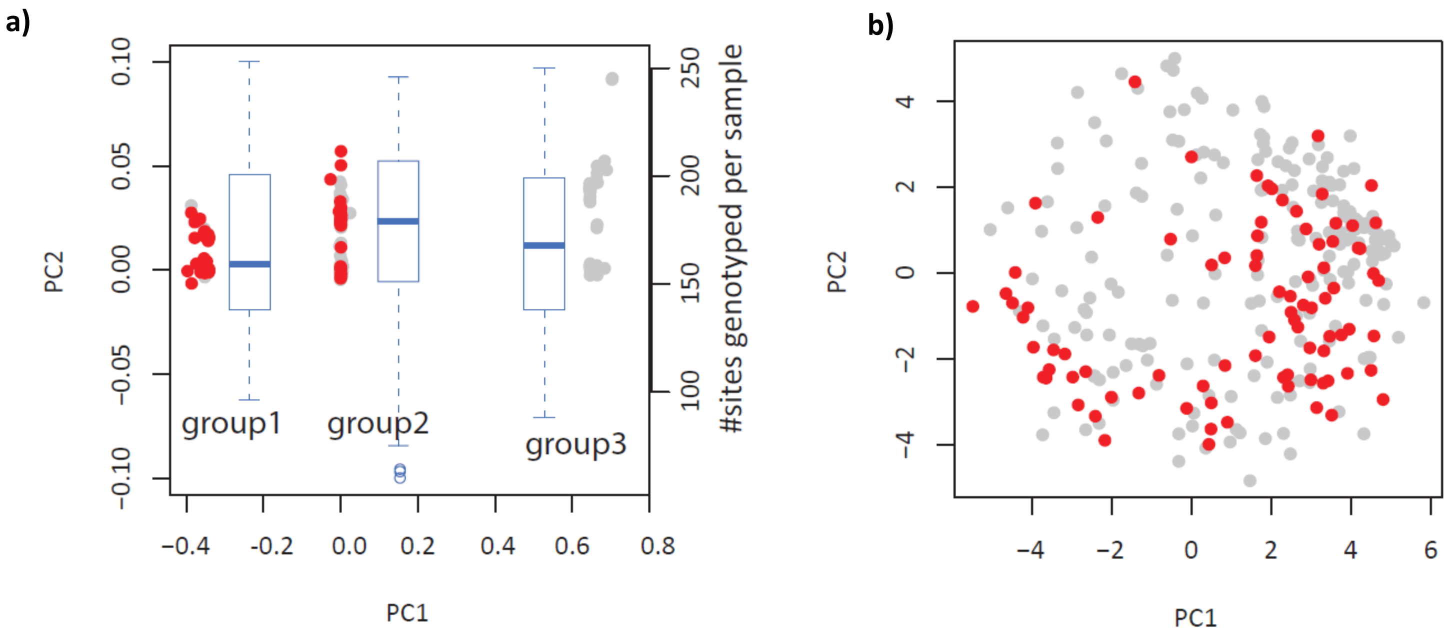


**Supplementary Figure 14:** **a**) PCA plot generated from highly represented 284 SNPs showing the boxplot on the side of each group, representing the distribution of number of sites genotyped per sample for that group. Samples divided into three groups by PC1 (n=100 for group 1, n=212 for group 2 , n=51 for group 3). All NAs in the genotype matrix were considered and replaced with the estimates by the pcaMethods package of R. For each boxplot the centre line depicts the median, box limits as upper and lower quartiles, whiskers as minimum and maximum values (that are not outliers). **b**) PCA plot if all the NAs in genotype matrix are replaced with 0.

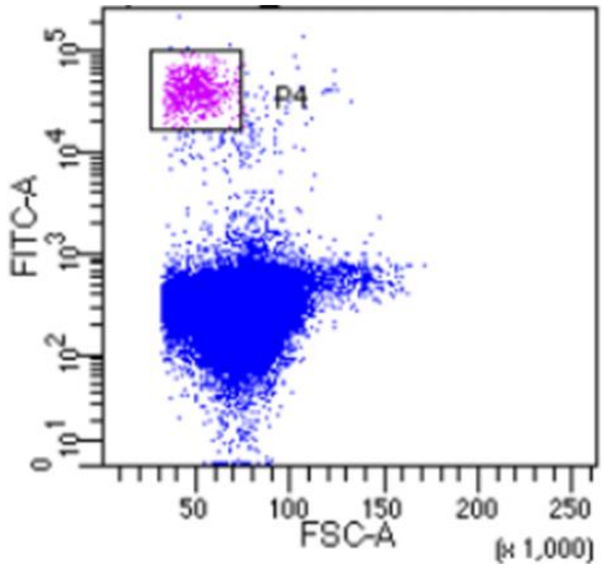


**Supplementary Figure 15:** A FACS dot plot showing the gating strategy for *P. falciparum* schizont culture stained with SYBR-Green dye (FITC channel).
