# Supplementary material for: Stochastic expression of invasion genes in Plasmodium falciparum schizonts
Source: Nat Commun. 2022 May 30;13:3004. doi: 10.1038/s41467-022-30605-z (PMC9151791; doi:10.1038/s41467-022-30605-z)
Supplement: Supplementary file 2 — Description of Additional Supplementary Information [file 41467_2022_30605_MOESM2_ESM.pdf]

Description of Additional Supplementary Files

File Name: Supplementary Data 1
Description: Differentially expressed genes along the pseudotemporal progression

File Name: Supplementary Data 2
Description: MPM pathways enrichment for differentially expressed genes along the pseudotemporal progression

File Name: Supplementary Data 3
Description: Variability index score for SCTS 1,4,5,6 and 7

File Name: Supplementary Data 4
Description: Highly variable genes for non-isogenic schizonts

File Name: Supplementary Data 5
Description: Highly variable genes for isogenic schizonts

File Name: Supplementary Data 6
Description:. qRT PCR Primer Sequences

File Name: Supplementary Data 7
Description: List of all 3459 SNPs

File Name: Supplementary Data 8
Description:. Genes associated with SNPs in isogenic cells
