# Supplementary material for: Stochastic expression of invasion genes in Plasmodium falciparum schizonts
Source: Nat Commun. 2022 May 30;13:3004. doi: 10.1038/s41467-022-30605-z (PMC9151791; doi:10.1038/s41467-022-30605-z)
Supplement: Supplementary file 11 — Reporting Summary [file 41467_2022_30605_MOESM11_ESM.pdf]

nature research

Corresponding author(s): Dr. Jaishree Tripathi

Last updated by author(s): Apr 8, 2022

Reporting Summary

Nature Research wishes to improve the reproducibility of the work that we publish. This form provides structure for consistency and transparency in reporting. For further information on Nature Research policies, see our Editorial Policies and the Editorial Policy Checklist.

Statistics

For all statistical analyses, confirm that the following items are present in the figure legend, table legend, main text, or Methods section.

n/a	Confirmed	
	x	The exact sample size (n) for each experimental group/condition, given as a discrete number and unit of measurement
x		A statement on whether measurements were taken from distinct samples or whether the same sample was measured repeatedly
	x	The statistical test(s) used AND whether they are one- or two-sided<br>*Only common tests should be described solely by name; describe more complex techniques in the Methods section.*
x		A description of all covariates tested
	x	A description of any assumptions or corrections, such as tests of normality and adjustment for multiple comparisons
	x	A full description of the statistical parameters including central tendency (e.g. means) or other basic estimates (e.g. regression coefficient) AND variation (e.g. standard deviation) or associated estimates of uncertainty (e.g. confidence intervals)
	x	For null hypothesis testing, the test statistic (e.g. F, t, r) with confidence intervals, effect sizes, degrees of freedom and P value noted<br>*Give P values as exact values whenever suitable.*
x		For Bayesian analysis, information on the choice of priors and Markov chain Monte Carlo settings
x		For hierarchical and complex designs, identification of the appropriate level for tests and full reporting of outcomes
x		Estimates of effect sizes (e.g. Cohen's d, Pearson's r), indicating how they were calculated

Our web collection on statistics for biologists contains articles on many of the points above.

Software and code

Policy information about availability of computer code

Data collection	Cells sorted using BD FACSDiva™ Software (version 6.0)
Data analysis	Trim Galore (version 0.6.6, https://www.bioinformatics.babraham.ac.uk/projects/trim_galore/)<br>ZEN 3.0 (blue edition ZEN lite)<br>In-house quality based read trimming script - Available upon request<br>Fastq-pair (https://github.com/linsalrob/fastq-pair)<br>Hisat2 (Kim et al., 2019)<br>Samtools (Li et al., 2007)<br>Bedtools (Quinlan et al., 2010)<br>GATK (McKenna et al., 2010)<br>Resampling-based sequential ensemble clustering (RSEC) - R package<br>ZINBWaVE - R package

For manuscripts utilizing custom algorithms or software that are central to the research but not yet described in published literature, software must be made available to editors and reviewers. We strongly encourage code deposition in a community repository (e.g. GitHub). See the Nature Research guidelines for submitting code & software for further information.

Data

Policy information about availability of data

All manuscripts must include a data availability statement. This statement should provide the following information, where applicable:

- Accession codes, unique identifiers, or web links for publicly available datasets
- A list of figures that have associated raw data
- A description of any restrictions on data availability

The data discussed in this publication have been deposited in NCBI's Gene Expression Omnibus (Edgar et al., 2002) and are accessible through GEO Series accession number GSE164459 (https://www.ncbi.nlm.nih.gov/geo/query/acc.cgi?acc=GSE164459)
The 3D7 reference transcriptome data can be accessed through GEO accession number GSE150484 (https://www.ncbi.nlm.nih.gov/geo/query/acc.cgi?acc=GSE150484).

Field-specific reporting

Please select the one below that is the best fit for your research. If you are not sure, read the appropriate sections before making your selection.

[x] Life sciences   [ ] Behavioural & social sciences   [ ] Ecological, evolutionary & environmental sciences

For a reference copy of the document with all sections, see nature.com/documents/nr-reporting-summary-flat.pdf

Life sciences study design

All studies must disclose on these points even when the disclosure is negative.

Sample size	No sample-size calculation was performed. We chose 295 non-isogenic parasites and 100 isogenic parasites for RNA sequencing as our data shows that a minimum of 100 cells is a close representation of the bulk parasite population (Spearman Correlation of ~ 0.7 to 0.8 to bulk parasite transcriptome - data not shown). Hence we chose a minimum of 100 cells (for example in isogenic cohort) for studying stochastic gene expression and draw conclusions representing the population.
Data exclusions	We filtered out transcriptomes having a significantly lower (3 time mad lower than median) mapping rate or total read counts; also samples were discarded if they presented <75% of the 269 highly appeared genes which were detected in 95% of PfRNA dilution samples.
Replication	Each 1-cell represents an independent biological sample from within a population for the RNA sequencing, qPCR and RNA-FISH experiments. Ten replicates of PfRNA dilutions were included with each batch of sequencing to measure technical noise within the dataset. Two biological replicates of non-isogenic schizonts and one biological replicate of isogenic schizonts were sequenced independently. For qPCR and RNA-FISH, multiple single schizonts from one biological replicate were studied. For comparison of MALBAC and SMARTseq2, 1-cell and 10-cell triplicates and thirty PfRNA dilutions were used to calculate correlation coefficients, mean, standard deviation and probability of detection (Figure 1 and Supplementary Figure 1). Each attempt of replication of PfRNA dilutions, 1-cells and 10-cells was successful for both MALBAC and SMARTseq2.
Randomization	Randomization is not relevant to this study as there are no subject or treatment groups involved. All non-isogenic cell samples belonged to a single cohort and were not allocated to multiple groups. During FACS sorting, cells are collected randomly from the pool of parasites, hence no randomization is needed.
Blinding	Blinding is not relevant to this study as there are no study participants/groups involved and no null hypothesis was tested. This is a exploratory RNAseq study.

Reporting for specific materials, systems and methods

We require information from authors about some types of materials, experimental systems and methods used in many studies. Here, indicate whether each material, system or method listed is relevant to your study. If you are not sure if a list item applies to your research, read the appropriate section before selecting a response.

Materials & experimental systems

n/a	Involved in the study	
[x]	[ ]	Antibodies
[ ]	[x]	Eukaryotic cell lines
[x]	[ ]	Palaeontology and archaeology
[x]	[ ]	Animals and other organisms
[x]	[ ]	Human research participants
[x]	[ ]	Clinical data
[x]	[ ]	Dual use research of concern

Methods

n/a	Involved in the study	
[x]	[ ]	ChIP-seq
[ ]	[x]	Flow cytometry
[x]	[ ]	MRI-based neuroimaging

Eukaryotic cell lines

Policy information about cell lines

Cell line source(s)	P. falciparum 3D7 strain was ordered from BEI Resource (Cat#MRA-102)
Authentication	None of the cell lines used was authenticated.
Mycoplasma contamination	All cell lines tested negative for mycoplasma.
Commonly misidentified lines (See ICLAC register)	Not applicable

Flow Cytometry

Plots

Confirm that:

- [x] The axis labels state the marker and fluorochrome used (e.g. CD4-FITC).
- [x] The axis scales are clearly visible. Include numbers along axes only for bottom left plot of group (a 'group' is an analysis of identical markers).
- [ ] All plots are contour plots with outliers or pseudocolor plots.
- [ ] A numerical value for number of cells or percentage (with statistics) is provided.

Methodology

Sample preparation	A sorbitol synchronised P. falciparum 3D7 schizont culture (40 to 44 HPI) was used for performing single cell sorting. 20 µl of infected RBCs (iRBC) were washed in 1X PBS twice and stained with SYBR green dye (final dilution 0.2 x) for 30 minutes at 37 oC in the dark. Next, the iRBC pellet was washed > 5 times with 1X PBS before final resuspension in 1X PBS solution ready for FACS. Uninfected RBCs were also stained in parallel as a control for gating strategy. iRBCs were sorted on BD FACSAria™ into 4 µl of lysis buffer (5x FS buffer = 100 µl, 0.1M DTT= 25 µl, 10mM dNTP= 25 µl, 40U/ul RNaseOUT= 5 µl, GAT-dT Primer (100 µM)= 12.5 µl, H2O = 331.5 µl, Triton X114= 1 µl) in 96 well plates, spinned briefly and processed immediately or stored at -80 oC for later.
Instrument	BD FACS Aria III Cell Sorter
Software	BD FACSDiva™ software (version 8.0.1)
Cell population abundance	The purity of infected red blood cell was confirmed by staining the culture with SYBR green dye (0.2X final concentration) for 30 minutes. An uninfected stained control was included to determine the gating strategy to exclude any uninfected red blood cells. Post sorting, Giemsa smear was made to confirm infected red blood cells.
Gating strategy	Uninfected red blood cells (RBCs) were SYBR green stained as a control to determine background fluorescence from uninfected RBCs. This was used to draw the gates for capturing SYBR green positive infected RBCs. Post sorting, Giemsa smear was made to confirm capturing of infected red blood cells.

- [x] Tick this box to confirm that a figure exemplifying the gating strategy is provided in the Supplementary Information.
